# Supplementary material for: Evidence of In Vitro Preservation of Human Nephrogenesis at the Single-Cell Level
Source: Stem Cell Reports. 2017 May 25;9(1):279–91. doi: 10.1016/j.stemcr.2017.04.026 (PMC5511042; doi:10.1016/j.stemcr.2017.04.026)
Supplement: Document S1. Supplemental Experimental Procedures, Figures S1–S4, and Tables S1–S3 [file mmc1.pdf]

Stem Cell Reports, Volume *9*

Supplemental Information

Evidence of In Vitro Preservation of Human Nephrogenesis at the Single-Cell Level

Naomi Pode-Shakked, Rotem Gershon, Gal Tam, Dorit Omer, Yehudit Gnatek, Itamar Kanter, Sarit Oriel, Guy Katz, Orit Harari-Steinberg, Tomer Kalisky, and Benjamin Dekel

EVIDENCE OF IN VITRO PRESERVATION OF HUMAN NEPHROGENESIS AT THE SINGLE CELL LEVEL

Pode-Shakked Naomi[1,2,3,7,8], Gershon Rotem[1,2,7,8], Tam Gal[4], Omer Dorit[1,2,] , Gnatek Yehudit[1,2], Kanter Itamar[4], Oriel Sarit[4], Katz Guy[1,2,3,5,7], Harari-Steinberg Orit[1,2], Kalisky Tomer[4,9], Dekel Benjamin[1,2,6,7, 9#]

[1]Pediatric Stem Cell Research Institute, Edmond and Lily Safra Children's Hospital, Sheba Medical Center, Tel-Hashomer, Israel

[2]Sheba Centers for Regenerative Medicine and Cancer Research, Sheba Medical Center, Tel-Hashomer, Israel

[3]The Dr. Pinchas Borenstein, Talpiot Medical Leadership Program, Sheba Medical Center, Tel-Hashomer, Israel

[4]Faculty of Engineering and Bar-Ilan Institute of Nanotechnology and Advanced Materials (BINA), Bar-Ilan University, Ramat Gan, Israel.

[5]The Joseph Buchman Gynecology and Maternity Center,Sheba Medical Center, Tel-Hashomer, Israel

[6]Division of Pediatric Nephrology, Edmond and Lily Safra Children's Hospital,

Sheba Medical Center, Tel-Hashomer, Israel

[7]Sackler Faculty of Medicine, Tel-AvivUniversity, Tel-Aviv, Israel

[8]The first two authors share first co-authorship

[9]The last two authors share senior co-authorship

Supplemental Information table of contents:

SUPPLEMENTAL FIGURES AND TABLES **3**

FIGURE S1| SIX2 AND EPCAM EXPRESSION IN HFK CELLS GROWN IN MNPEM REVEALS CHARACTERISTIC "NICHES" 3
FIGURE S2| FACS ANALYSIS OF HFK ACCORDING TO NCAM, CD133, AND EPCAM EXPRESSION LEVELS SHOWS THREE CELL POPULATIONS AT DIFFERENT STAGES OF DIFFERENTIATION WITHIN THE NCAM+CD133- FRACTION 4
FIGURE S3| IDENTIFICATION OF THE SECOND (NON CAP) MESENCHYMAL CELL POPULATION. SINGLE CELL QPCR GENE EXPRESSION ANALYSIS OF HFK CELLS GROWN IN SFM AND SORTED ACCORDING TO NCAM AND CD133 SHOWS A PREDOMINANTLY EPITHELIAL PHENOTYPE 5
FIGURE S4| NCAM+CD133-EPCAM- CELLS GROWN IN MNPEM PRESERVE SIX2 EXPRESSION AND CAN DIFFERENTIATE TO PROXIMAL AND DISTAL TUBULAR EPITHELIA. FOXD1 IS DOWNREGULATED IN HFK CELLS GROWN IN MNPEM. 7
TABLE S1| 48 TAQMAN GENE EXPRESSION ASSAYS USED FOR "BULK" MICROFLUIDIC MULTIPLEXED QPCR 8
TABLE S2| AVERAGE PERCENTAGES (N=3) OF NEPHROGENIC RENAL MET COMPARTMENTS ACCORDING TO NCAM, CD133, AND EPCAM IN FRESH HFK AND HFK GROWN IN MNPEM, SCM AND SFM. 10
TABLE S3| 48 TAQMAN GENE EXPRESSION ASSAYS USED FOR SINGLE CELL MICROFLUIDIC MULTIPLEXED QPCR 11

SUPPLEMENTAL EXPERIMENTAL PROCEDURES **13**

IF STAINING OF CELLS 13
IN VIVO WT XENOGRAFT FORMATION 13
SINGLE CELL GENE EXPRESSION ANALYSIS 13
STATISTICAL ANALYSIS 14

REFERENCES **15**

Supplemental figures and tables

Figure S1| SIX2 and EpCAM expression in hFK cells grown in mNPEM reveals characteristic "niches"

A

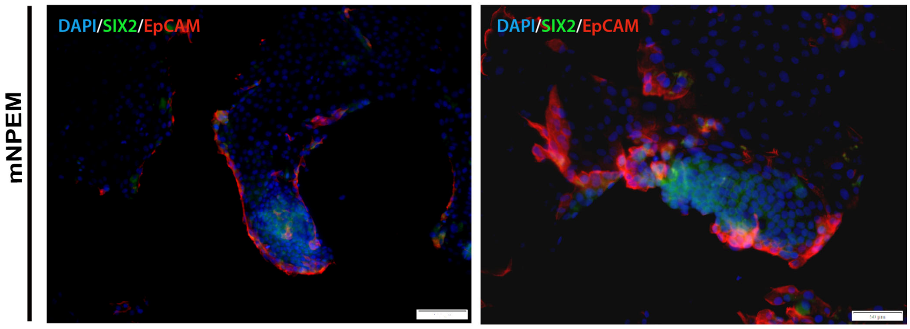

B

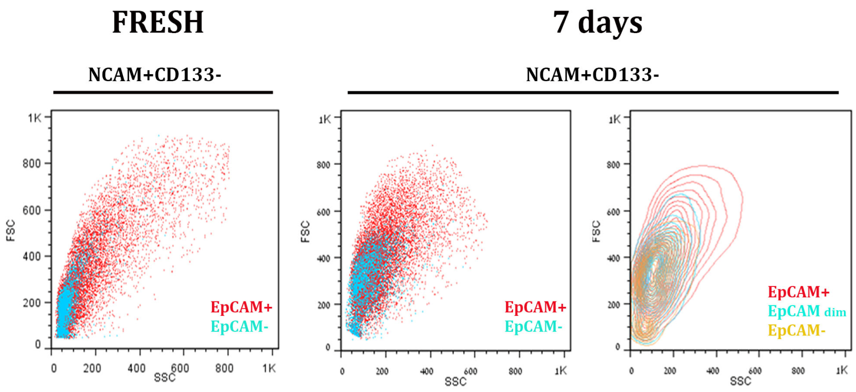

(A) Double labeling of hFK cells grown in mNPEM for SIX2 and EpCAM reveals a unique "niche" microstructure characterized by small inner cells that positively stain for SIX2 and peripheral larger epithelial cells that positively stain for EpCAM. Images were obtained using Olympus DP72 camera attached to Olympus BX51 fluorescence microscope and processed via cell Sens standard software. Bar represents 100µm in left panel and 50µm in right panel; (B) Representative FACS analysis of Fresh hFK and hFK cultured in mNPEM for 7 days based on forward and side scatter (FSC/SSC) plot. Close examination of the different cell subpopulations according to EpCAM distribution within the NCAM1+CD133- cell fraction shows EpCAM- (as well as EpCAM-/dim) cells in both Fresh hFK and mNPEM cultured hFK to possess low FSC and SSC parameters relative to EpCAM+ (as well as EpCAMbright), in accordance with their small size. These findings demonstrate that similar cellular diversity is found in both mNPEM-grown hFK cells and in fresh uncultured hFK cells. (Related to Figure 1)

Figure S2| FACS analysis of hFK according to NCAM, CD133, and EpCAM expression levels shows three cell populations at different stages of differentiation within the NCAM+CD133- fraction

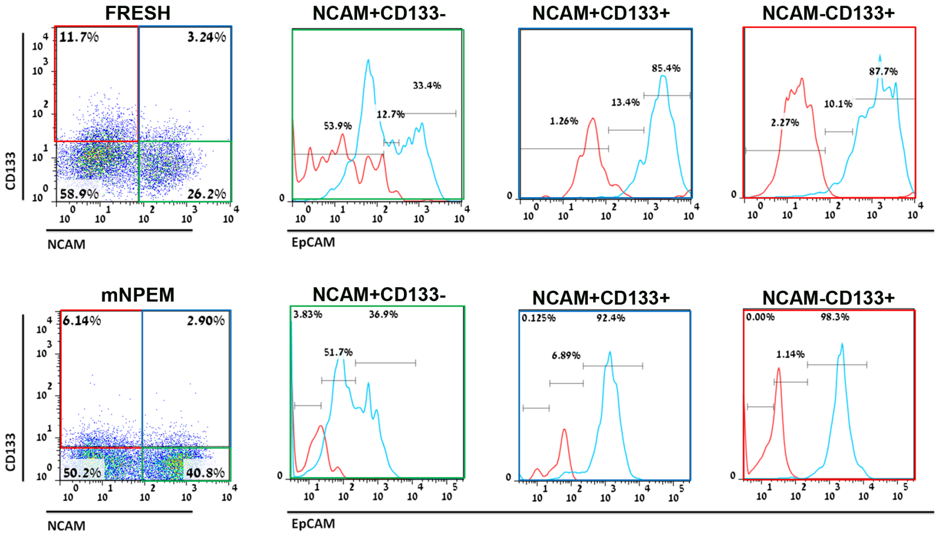

Representative FACS analysis of Fresh hFK and hFK cultured in mNPEM for 7 days (3rd hFK replicate). Cells grown in mNPEM preserves the NCAM1^{+}CD133^{-} cell fraction in a way that is comparable to fresh cells (~26% and 40% respectively). Within the NCAM1^{+}CD133^{-} cell fraction most cells are EpCAM^{dim} or EpCAM^{-} in both Fresh hFK and hFK cultured in mNPEM (53.9+12.7=66.6% and 3.83+51.7=55.53% respectively). Moreover, the NCAM1^{+}CD133^{+} and NCAM^{-} CD133^{+} subpopulations show gradual increase in EpCAM^{bright} cells (92% and 98% respectively) in mNPEM grown cells. This is consistent with earlier findings and provides further support to our hypothesis that all hFK renal MET epithelial lineages are preserved in mNPEM (see also Figure 2C). (Related to Figure 2)

Figure S3| Identification of the second (non Cap) mesenchymal cell population. Single cell qPCR gene expression analysis of hFK cells grown in SFM and sorted according to NCAM and CD133 shows a predominantly epithelial phenotype

A

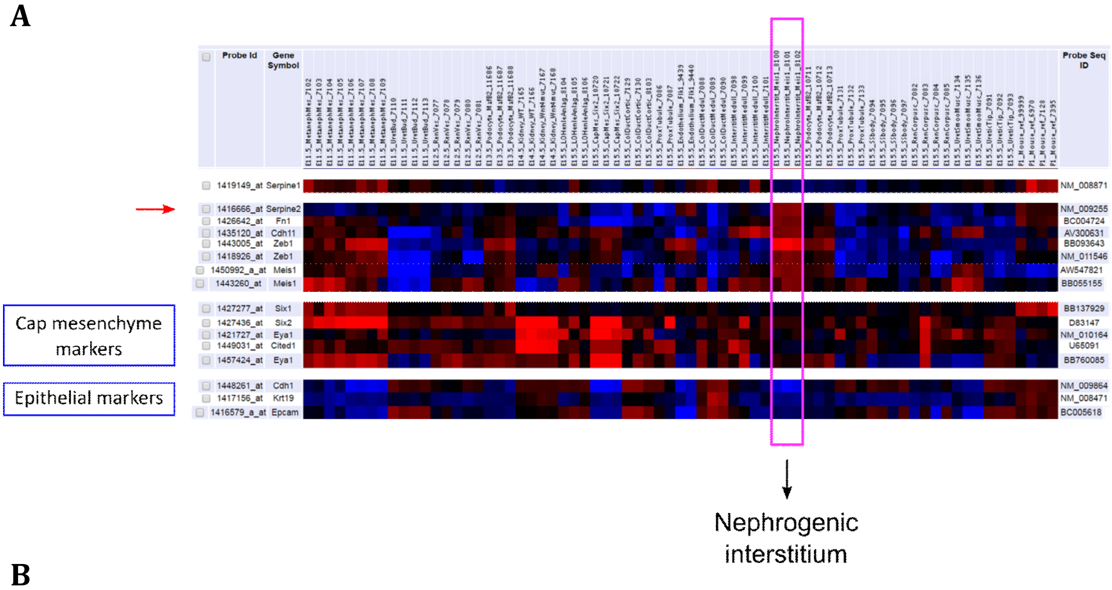

B

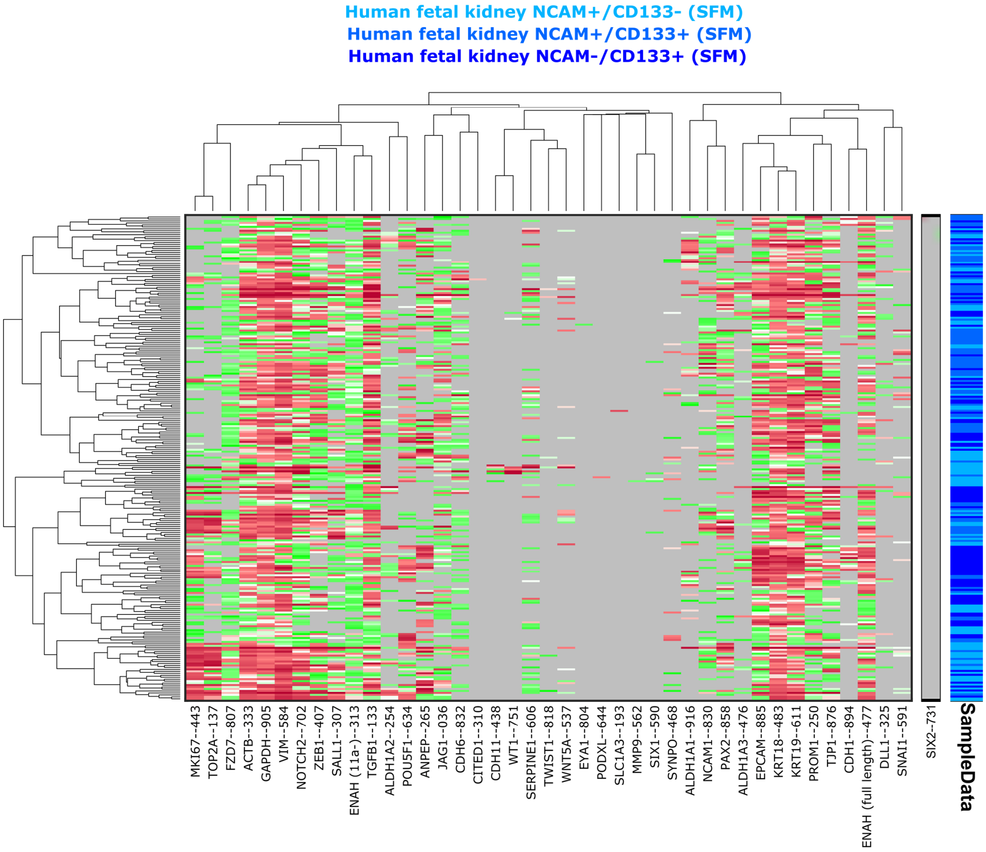

(A) Identification of the second (non-Cap) human mesenchymal cell population in hFK cells grown in mNPEM. Shown are microarray expression measurements queried from the GUDMAP database (http://www.gudmap.org). It can be seen that the nephrogenic interstitial (Meis1+) cell population in E15.5 mouse embryos [1] behaves similar to the second human non-Cap mesenchymal cell population, with SERPINE2 – a paralog of SERPINE1 – being over-expressed along with CDH11 and ZEB1. The Cap-mesenchyme markers (SIX1, SIX2, EYA1, and CITED1) and epithelial markers (CDH1, KRT19, and EpCAM) are relatively under-expressed in this population. We therefore hypothesize that the second non-Cap mesenchymal cell subpopulation (in hFK cells grown in mNPEM) represents the nephrogenic interstitium, and that SERPINE2 in mice interchanges with its paralog SERPINE1 in humans as a marker for the nephrogenic interstitial cells. (B) A gene expression heat map of ~240 single cells (rows) and 42 genes (columns) measured simultaneously from each cell. Gene expression levels (in terms of threshold cycles, Ct) were standardized and clustered such that phenotypically similar cells are grouped next to each other (Red – high expression, Green – low expression, Gray – no expression). Cells were isolated from human fetal kidney, cultured in SFM for 7 days, and sorted by FACS into 3 fractions: NCAM+CD133-, NCAM+CD133+, and NCAM-CD133+. The colors in the column on the right of the heatmap represent the FACS-sorted fraction-of-origin of each individual cell. It can be seen that all fractions have a predominantly epithelial phenotype (EPCAM+KRT18+KRT19+SIX2-). Although in the NCAM+CD133- fraction we did find a small number of more early mesenchymal cells (CDH11+), no measurable SIX2+ cells within the ~240 cells that we measured. Genes that had zero expression in all cells were excluded from the heatmap (apart from SIX2) (Related to Figure 3).

Figure S4| NCAM+CD133-EpCAM- cells grown in mNPEM preserve SIX2 expression and can differentiate to proximal and distal tubular epithelia. FOXD1 is downregulated in hFK cells grown in mNPEM.

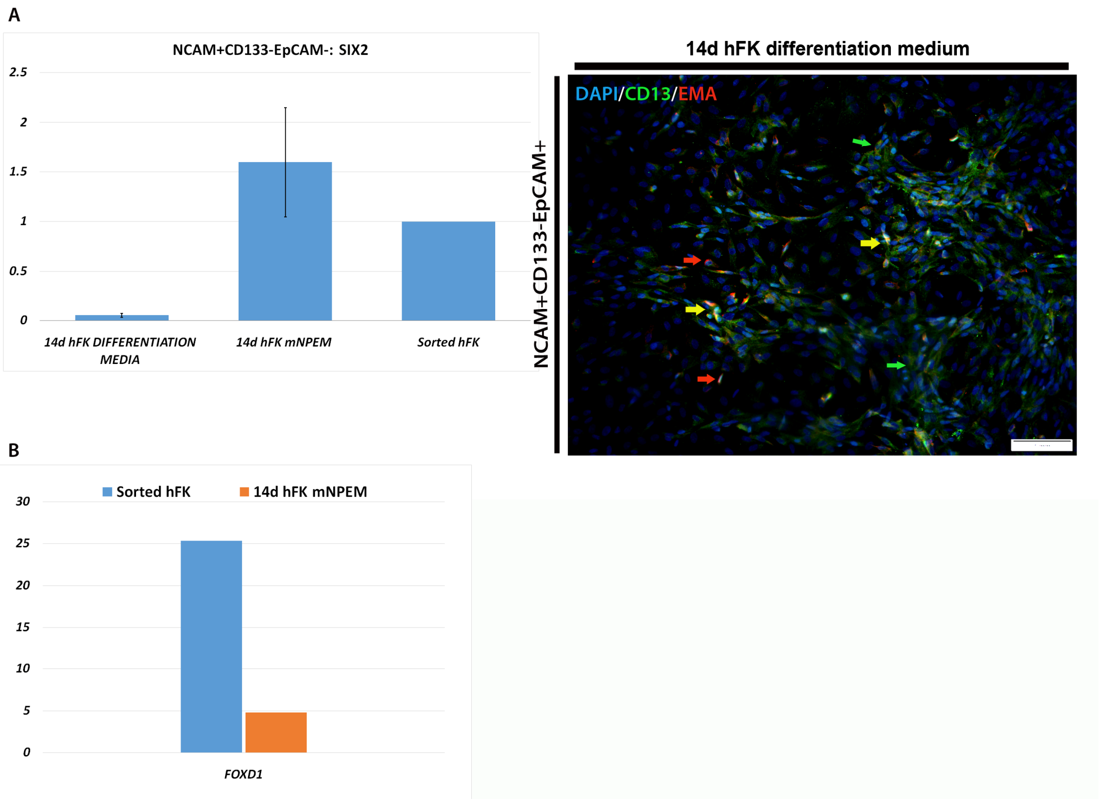

(A) Left panel: qRT-PCR analysis of SIX2 expression in hFK cells grown in mNPEM. NCAM1$^+$CD133$^-$EpCAM$^-$ cells cultured in mNPEM showed preservation of SIX2 expression after 14 days in culture that is comparable to freshly sorted hFK cells. In contrast, cells grown in a differentiation media show drastic downregulation of SIX2 expression. Note that the values for NCAM1$^+$CD133$^-$EpCAM$^-$ freshly sorted hFK cells were used as reference for normalization (therefore=1) and all other values were calculated with respect to them. Experiments were performed on hFK from 2 sources (n=2). Results are presented as the mean±S.E.M of three separate experiments; *$p<0.05$; Right panel: Immunofluorescence staining of NCAM1$^+$CD133$^-$EpCAM$^-$ cells grown in a differentiation media for 14 days for CD13 (a proximal tubule marker, green) and EMA (a distal tubule marker, red) demonstrating several stages of renal MET of human nephrogenesis by cells expressing either CD13 (green arrow) or EMA (red arrow). Double labeling for both CD13 and EMA for a small number of cells suggests an earlier intermediate state of epithelial differentiation (yellow arrow). (B) qRT-PCR analysis of FOXD1 expression in sorted hFK cells grown in mNPEM reveals dramatic downregulation in FOXD1 expression levels at day 14 suggesting the reduction of stromal elements (FOXD1$^+$) compared to T0. (Related to Figure 1).

Table S1| 48 Taqman gene expression assays used for "bulk" microfluidic multiplexed qPCR

	Assay ID	Gene Symbol
1	Hs00357333_g1	ACTB
2	Hs00946916_m1	ALDH1A1
3	Hs00174265_m1	ANPEP
4	Hs00166067_m1	AQP1
5	Hs00185020_m1	AQP3
6	Hs00984230_m1	B2M
7	Hs01023894_m1	CDH1
8	Hs00191832_m1	CDH6
9	Hs00366310_m1	CITED1
10	Hs00153607_m1	CUBN
11	Hs01011325_g1	DLL1
12	Hs00901885_m1	EPCAM
13	Hs00559473_s1	FOXC1
14	Hs00270117_s1	FOXD1
15	Hs99999905_m1	GAPDH
16	Hs00735523_m1	IRX3
17	Hs01070036_m1	JAG1
18	Hs00969422_m1	LGR5
19	Hs00189742_m1	LRP2
20	Hs01032443_m1	MKI67
21	Hs00410317_m1	MUC1
22	Hs00941830_m1	NCAM1
23	Hs01050702_m1	NOTCH2

24	Hs00190446_m1	NPHS1
25	Hs00377071_m1	OSR1
26	Hs00240858_m1	PAX2
27	Hs00248563_m1	PLCB1
28	Hs01574644_m1	PODXL
29	Hs01009250_m1	CD133
30	Hs00230907_m1	RARA
31	Hs00231307_m1	SALL1
32	Hs01548617_m1	SCNN1B
33	Hs00232731_m1	SIX2
34	Hs00165731_m1	SLC12A1
35	Hs01027568_m1	SLC12A3
36	Hs01573790_m1	SLC5A1
37	Hs00195591_m1	SNAI1
38	Hs00950344_m1	SNAI2
39	Hs00998133_m1	TGFB1
40	Hs01032137_m1	TOP2A
41	Hs01675818_s1	TWIST1
42	Hs00358451_m1	UMOD
43	Hs00185584_m1	VIM
44	Hs00260769_m1	WNK4
45	Hs01055707_m1	WNT3A
46	Hs00229142_m1	WNT4
47	Hs00998537_m1	WNT5A
48	Hs01103751_m1	WT1

(Related to Figure 1)

Table S2| Average percentages (n=3) of nephrogenic renal MET compartments according to NCAM, CD133, and EpCAM in fresh hFK and hFK grown in mNPEM, SCM and SFM.

	FRESH	mNPEM	SCM	SFM
NCAM+CD133-	20.05	20.33	4.00	3.02
NCAM+CD133-EPCAM-	5.51	7.42	0.14	0.13
NCAM+CD133-EPCAMdim	6.82	5.74	0.98	0.42
NCAM+CD133-EPCAMbright	6.92	7.52	2.88	2.45

(Related to Figure 2)

Table S3| 48 Taqman gene expression assays used for single cell microfluidic multiplexed qPCR

	Assay ID	Gene symbol
1	Hs00357333_g1	ACTB
2	Hs00946916_m1	ALDH1A1
3	Hs00180254_m1	ALDH1A2
4	Hs00167476_m1	ALDH1A3
5	Hs00174265_m1	ANPEP
6	Hs01023894_m1	CDH1
7	Hs00156438_m1	CDH11
8	Hs00191832_m1	CDH6
9	Hs00366310_m1	CITED1
10	Hs01011325_g1	DLL1
11	Hs04260477_m1	ENAH (full length)
12	Hs00983313_g1	ENAH (11a-)
13	Hs00901885_m1	EPCAM
14	Hs00166804_m1	EYA1
15	Hs00942807_s1	FZD7
16	Hs99999905_m1	GAPDH
17	Hs01070036_m1	JAG1
18	Hs02827483_g1	KRT18
19	Hs01051611_gH	KRT19
20	Hs00412974_m1	MFAP4
21	Hs01032443_m1	MKI67
22	Hs00957562_m1	MMP9
23	Hs00410317_m1	MUC1

24	Hs00941830_m1	NCAM1
25	Hs01050702_m1	NOTCH2
26	Hs00377071_m1	OSR1
27	Hs00240858_m1	PAX2
28	Hs01574644_m1	PODXL
29	Hs00999634_gH	POU5F1
30	Hs01009250_m1	CD133
31	Hs00231307_m1	SALL1
32	Hs00413788_m1	SALL2
33	Hs01126606_m1	SERPINE1
34	Hs00195590_m1	SIX1
35	Hs00232731_m1	SIX2
36	Hs00188193_m1	SLC1A3
37	Hs00195591_m1	SNAI1
38	Hs00950344_m1	SNAI2
39	Hs00702468_s1	SYNPO
40	Hs00998133_m1	TGFB1
41	Hs01551876_m1	TJP1
42	Hs01032137_m1	TOP2A
43	Hs01675818_s1	TWIST1
44	Hs00185584_m1	VIM
45	Hs00229142_m1	WNT4
46	Hs00998537_m1	WNT5A
47	Hs01103751_m1	WT1
48	Hs01566407_m1	ZEB1

(Related to Figure 3)

Supplemental experimental procedures

IF staining of cells

Cells were fixed with 4% PFA in PBS for 10 min, and washed with PBS–Tween (0.05%). Then, the cells were blocked with Cas-Block solution for 1 h at RT followed by incubation with primary antibodies for SIX2 (11562-1-AP, Proteintech), EpCAM (CBL251,Millipore), CD13 (Sigma), and EMA (Cell Marque) for 1h in room temperature. Cells were then washed and then incubated with a secondary antibody for 1h in room temperature. Following PBS-Tween washes, mounting containing DAPI (DapiFluoromount-G; SouthernBiotech, 0100-20) was applied. Images were obtained by Olympus BX51TF fluorescence microscope using Olympus DP72 camera and cellSens standard software. For comparative analysis between the fluorescence signal of SIX2 cells, images were analyzed using ImageJ software. Signal quantification was calculated for each nucleus by determining its area and quantification of the signal in that area. The corrected total cell fluorescence (CTCF) was calculated as: integrated density − (area of selected cell × mean fluorescence of background reading).

In vivo WT xenograft formation

All animal experiments were conducted in accordance with the National Institutes of Health guidelines for the care and use of animals and with an approved animal protocol from the Sheba medical center Animal Care and Use Committee. Initial WT xenografting to 5-8 weeks old, female, nonobese diabetic immune-deficient mice was performed as previously described [2]. Briefly, primary WT tissue was cut into 2-5mm pieces and implanted subcutaneously in the back of the mouse. Tumors were harvested approximately 3-6 month post implantation or when they reached a size of 1.5cm diameter. Single cells suspensions were obtained by mincing the samples in Iscove's modification of Dulbecco's medium (IMDM) containing antibiotics (penicillin and streptomycin), followed by treatment with collagenase IV for 2h at 37◦C. After addition of IMDM at twice the volume of the collagenase solution, the enzymatically treated tissue was triturated and the suspension was filtered using a 100µm cell strainer and washed twice with IMDM containing antibiotics. Erythrocytes were removed by ACK RBS lysis buffer.

Single cell gene expression analysis

We combined single cell expression data from the following biological samples:

1. Human fetal kidney no. 104 (hFK104): Cells isolated from a human fetal kidney, cultured for 7 days in NPEM for a single passage, and sorted by FACS into 3 fractions: $NCAM^{+}CD133^{-}EpCAM^{-}$, $NCAM^{+}CD133^{-}EpCAM^{dim}$, and $NCAM^{+}CD133^{+}$.

2. Human fetal kidney no. 103 (hFK103): Cells isolated from a human fetal kidney, cultured for 7 days in SFM for a single passage, and sorted by FACS into 3 fractions: $NCAM^{+}CD133^{-}$, $NCAM^{+}CD133^{+}$, $NCAM^{-}CD133^{+}$. Additionally, a 4th fraction of non-gated cells (ignoring markers) was sorted.

3. Wilms' tumor no. 11 – a patient-derived xenograft (W011 PDX): A late passage blastemal Wilms' tumor patient-derived xenograft. After 10 passages in NOD-SCID mice, cells were cultured for 4 days in SCM, and sorted into 2 fractions: $NCAM^{+}ALDH1^{+}$ (the tumorigenic fraction) and $NCAM^{+}ALDH1^{-}$ (the non-tumorigenic fraction).

Statistical analysis

Results are expressed as the mean ± S.E.M, unless otherwise indicated. Statistical differences in gene expression between hFK cell populations were evaluated using the non-parametric, one sided Sign test. Statistical differences between additional data groups were determined with Student's t test. For all statistical analysis, the level of significance was set as $p<0.05$ unless otherwise indicated. All experiments were performed in duplicates and on at least 3 tissue sources (n=3).

References

1. Brunskill EW, A.B., Georgas K, Rumballe B, Valerius MT, Aronow J, et al. , *Atlas of Gene Expression in the Developing Kidney at Microanatomic Resolution. .* Dev Cell., 2008. **15**: p. 781–791.

2. Dekel, B., et al., Multiple imprinted and stemness genes provide a link between normal and tumor progenitor cells of the developing human kidney. Cancer Res, 2006. **66**(12): p. 6040-9.
