# Supplementary material for: Evidence of In Vitro Preservation of Human Nephrogenesis at the Single-Cell Level
Source: Stem Cell Reports. 2017 May 25;9(1):279–91. doi: 10.1016/j.stemcr.2017.04.026 (PMC5511042; doi:10.1016/j.stemcr.2017.04.026)
Supplement: Document S2. Article plus Supplemental Information [file mmc2.pdf]

Stem Cell Reports
Article

OPEN ACCESS

Evidence of In Vitro Preservation of Human Nephrogenesis at the Single-Cell Level

Naomi Pode-Shakked,[1,2,3,7,8] Rotem Gershon,[1,2,7,8] Gal Tam,[4] Dorit Omer,[1,2] Yehudit Gnatek,[1,2] Itamar Kanter,[4] Sarit Oriel,[4] Guy Katz,[1,2,3,5,7] Orit Harari-Steinberg,[1,2] Tomer Kalisky,[4,9] and Benjamin Dekel[1,2,6,7,9,*]

[1]Pediatric Stem Cell Research Institute, Edmond and Lily Safra Children's Hospital
[2]Sheba Centers for Regenerative Medicine and Cancer Research
[3]The Dr. Pinchas Borenstein Talpiot Medical Leadership Program
Sheba Medical Center, Tel-Hashomer 52621, Israel
[4]Faculty of Engineering and Bar-Ilan Institute of Nanotechnology and Advanced Materials (BINA), Bar-Ilan University, Ramat Gan 5290002, Israel
[5]The Joseph Buchman Gynecology and Maternity Center, Sheba Medical Center, Tel-Hashomer 52621, Israel
[6]Division of Pediatric Nephrology, Edmond and Lily Safra Children's Hospital, Sheba Medical Center, Tel-Hashomer 52621, Israel
[7]Sackler Faculty of Medicine, Tel-Aviv University, Tel-Aviv 6997801, Israel
[8]Co-first author
[9]Co-senior author
*Correspondence: binyamin.dekel@sheba.health.gov.il
http://dx.doi.org/10.1016/j.stemcr.2017.04.026

SUMMARY

During nephrogenesis, stem/progenitor cells differentiate and give rise to early nephron structures that segment to proximal and distal nephron cell types. Previously, we prospectively isolated progenitors from human fetal kidney (hFK) utilizing a combination of surface markers. However, upon culture nephron progenitors differentiated and could not be robustly maintained in vitro. Here, by culturing hFK in a modified medium used for in vitro growth of mouse nephron progenitors, and by dissection of $NCAM^{+}$/$CD133^{-}$ progenitor cells according to EpCAM expression ($NCAM^{+}/CD133^{-}/EpCAM^{-}$, $NCAM^{+}/CD133^{-}/EpCAM^{dim}$, $NCAM^{+}/CD133^{-}/EpCAM^{bright}$), we show at single-cell resolution a preservation of uninduced and induced cap mesenchyme as well as a transitioning mesenchymal-epithelial state. Concomitantly, differentiating and differentiated epithelial lineages are also maintained. In vitro expansion of discrete stages of early human nephrogenesis in nephron stem cell cultures may be used for drug screening on a full repertoire of developing kidney cells and for prospective isolation of mesenchymal or epithelial renal lineages for regenerative medicine.

INTRODUCTION

Nearly 26 million Americans, one in every nine, harbor kidney disease (Trivedi, 2010). Despite recent medical advances, treatment options for patients with renal failure are limited. The alternatives available to patients who succumb to terminal renal disease are either supportive treatment in the form of dialysis or whole organ replacement by kidney transplantation. Dialysis is associated with long-term morbidity, mortality, and poor quality of life. The shortage of donor organs and the long wait time on the recipient list hamper renal transplantation (Daar, 2006). The number of patients with terminal renal disease has increased, and the treatment costs for these patients now exceed the cumulative costs of treating cancer patients (Trivedi, 2010). Due to the growing number of patients with kidney disease and the limited treatment options, alternative treatments are clearly in need.

Various types of stem cells may be applicable as a platform for cell therapy for renal disease (Pleniceanu et al., 2010; Harari-Steinberg et al., 2011). Nevertheless, we now know that (1) bone marrow and blood stem cells do not generate nephron cell types (Duffield et al., 2005; Krause and Cantley, 2005; Dekel et al., 2006a) and (2) no adult kidney epithelial stem cell with wide nephrogenic potential exists in the adult kidney (Rinkevich et al., 2014). Hence, isolation of tissue stem/progenitor cells from fetal kidneys is an attractive option for replenishment of nephron cells (Pleniceanu et al., 2010; Harari-Steinberg et al., 2011). The mammalian kidney is formed via reciprocally inductive interactions between two mesoderm precursor tissues, the metanephric mesenchyme (MM) and the ureteric bud (UB) (Pleniceanu et al., 2010). In response to UB signals, induced MM cells acquire an epithelial phenotype (mesenchymal to epithelial transition; MET) to generate committed nephron progenitor populations and sequentially form pre-tubular aggregates, renal vesicles, and C- and S-shaped bodies that eventually expand to give rise to mature nephrons (Pleniceanu et al., 2010). Recent lineage-tracing experiments of cell populations in transgenic mouse models have established that the transcription factor SIX2 signifies a multipotent progenitor cell subpopulation in the MM that condenses to form the cap mesenchyme (CM) around the UB, and is capable of self-renewing and differentiating toward different types

This is an open access article under the CC BY license (http://creativecommons.org/licenses/by/4.0/).

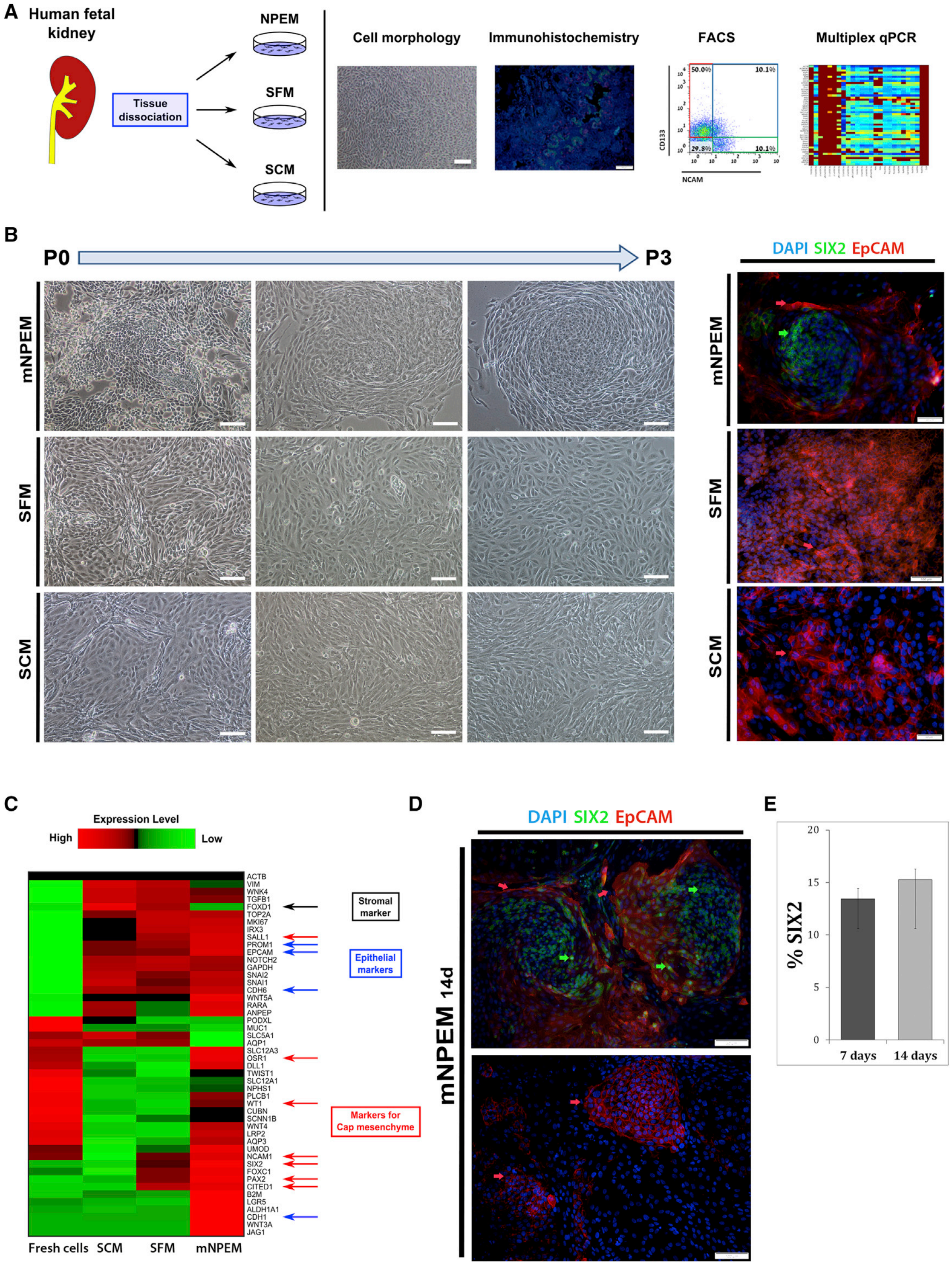

Figure 1. In Vitro Growth of hFK Cells in Monolayer Preserves Diversity of Human Embryonic Kidney Cells Only in mNPEM

(A) Schematic representation of experiments performed.

(B) (Left) Representative morphology of hFK cells cultured in SCM, SFM, or mNPEM over 3 passages. In mNPEM, heterogeneous cell morphology is observed during in vitro culturing on MG-coated plates showing cobblestone, spindle-shaped, and small ovoid-like cells. In addition, cells are organized in unique niches. In contrast, hFK cells grown in SFM show mostly cobblestone morphology that is consistent

(legend continued on next page)

of nephron epithelia (Boyle et al., 2008; Kobayashi et al., 2008; O'Brien et al., 2016).

Nevertheless, only a few studies have utilized human fetal kidney (hFK) as starting material for regenerative purposes (Harari-Steinberg et al., 2013). More than a decade ago, we started utilizing hFK for tissue transplantation and in vivo organogenesis (Dekel et al., 1997, 2002, 2003). We then continued with derivation of specific hFK cell types suitable for in vitro manipulation/expansion and cell therapy (Dziedzic et al., 2014). Hypothesizing that the blastema in human Wilms' tumor represents a transformed hFK CM, we concomitantly profiled blastema-enriched human Wilms' tumors, which contain numerous undifferentiated renal progenitors, along with human fetal kidneys, and discovered progenitor biomarkers on the cell surface, allowing for sorting of human developmental renal precursors (Harari-Steinberg et al., 2013; Dekel et al., 2006b; Metsuyanim et al., 2009; Pode-Shakked et al., 2016). Importantly, we showed that the latter can be useful for cell replacement and functional repair of chronic kidney injury in mice (Harari-Steinberg et al., 2013). For functional studies we have used hFK NCAM1$^+$ cells that contain the CM stage and early nephron differentiation, and hence are a heterogeneous cell subset. With the goal of minimizing heterogeneity, we have recently better pinpointed early stages of human nephrogenesis with a combination of surface markers (NCAM1$^+$CD133$^-$), allowing for isolation of hFK SIX2-expressing cells (Pode-Shakked et al., 2016). Nevertheless, early nephrogenesis and especially human CM SIX2$^+$ cells were only minimally represented following sorting attempts, as placing hFK cells in either serum-containing medium (SCM) or serum-free medium (SFM) resulted in dedifferentiation or epithelial differentiation, respectively, with resultant loss of the human CM early on. Thus, the expansion of hFK prior to prospective isolation depleted, at least in part, crucial nephrogenic cell types. It is noteworthy that while the human CM needs to be maintained in vitro, the human wild-type (WT) blastema can be readily propagated in vivo as human xenografts, allowing for constant isolation of WT stem/progenitors (Shukrun et al., 2014). A recent breakthrough in culturing mouse CM and the derived SIX2$^+$ cells (Brown et al., 2015; ýLi et al., 2016) has allowed us to modify the hFK culture and show robust maintenance of a human CM and a SIX2-expressing fraction in sorted hFK cell subpopulations. Accordingly, a combination of hFK expansion, fluorescence-activated cell sorting (FACS) analysis, and measurements of gene expression in single cells of sorted hFK fractions showed for the first time a preservation in culture of both the mesenchymal and epithelial cell repertoires engaged in human nephrogenesis.

RESULTS

In Vitro Growth of hFK Cells in Monolayer Preserves Diversity of Human Embryonic Kidney Cells Only in mNPEM

We have previously managed to grow hFK in SFM as monolayer preserving CM elements for a short time period (Harari-Steinberg et al., 2013; Pode-Shakked et al., 2016). However, preservation of MET of human nephrogenesis in vitro has not been established until now (Figure 1A). Using three different media, namely SCM, SFM, and modified

with epithelial differentiation, and SCM cultures show spindle-shaped morphology that is becoming predominant as the passage number increases. Cells were photographed using a Nikon Digital Sight camera attached to a Nikon Eclipse TS100 microscope. Scale bars, 100 μm.
(Right) Immunofluorescence staining for SIX2 and EpCAM in hFK cultures shows that the described heterogeneous "niches" appear only in cells grown in mNPEM (upper image), but not in SFM or SCM. The inner part of the niche contains small cells that are positive for SIX2 while the periphery of the niche is composed mainly of EpCAM$^+$ epithelial cells. In contrast, for both SFM and SCM, no SIX2$^+$ cells could be found and the cells are predominantly EpCAM$^+$ (middle and lower images). Images were obtained using an Olympus DP72 camera attached to an Olympus BX51 fluorescence microscope and processed via cellSens standard software. Scale bars, 50 μm (upper panel) and 100 μm (middle and lower panels).
(C) A heatmap representing gene expression levels that were obtained from microfluidic multiplex qPCR of "bulk" RNA from freshly dissociated hFK cells and for hFK cells grown in mNPEM, SFM, or SCM for 7 days. Cells grown in mNPEM preserve diverse hFK lineages, as can be seen from high expression of the markers for CM (SIX2, PAX2, CITED1, SALL1, OSR1, and WT1) and differentiated nephron epithelia (EPCAM, CDH6, and CDH1), as well as low expression of stromal associated genes (FOXD1). In contrast, cells grown in SFM and SCM show significantly lower expression levels for CM markers (at least 5-fold downregulation for SIX2, 15-fold for OSR1, and 25-fold for CITED1).
(D) Double labeling of hFK cells grown in mNPEM for 14 days for SIX2 and EpCAM. The upper image represents the "niche" phenotype characterize by inner cells positively stained for SIX2 (green arrows) and peripheral epithelial cells positive for EpCAM (red arrows). The lower image represents a late epithelial stage of human nephrogenesis by cells that predominantly express only EpCAM. Images were obtained using an Olympus DP72 camera attached to an Olympus BX51 fluorescence microscope and processed via cellSens standard software. Scale bars, 100 μm.
(E) Bar graph representing the percentage of SIX2$^+$ cells from hFK cells grown in mNPEM compared with fresh hFK (data were averaged over n = 3 independent experiments).
See also Figures S1 and S4.

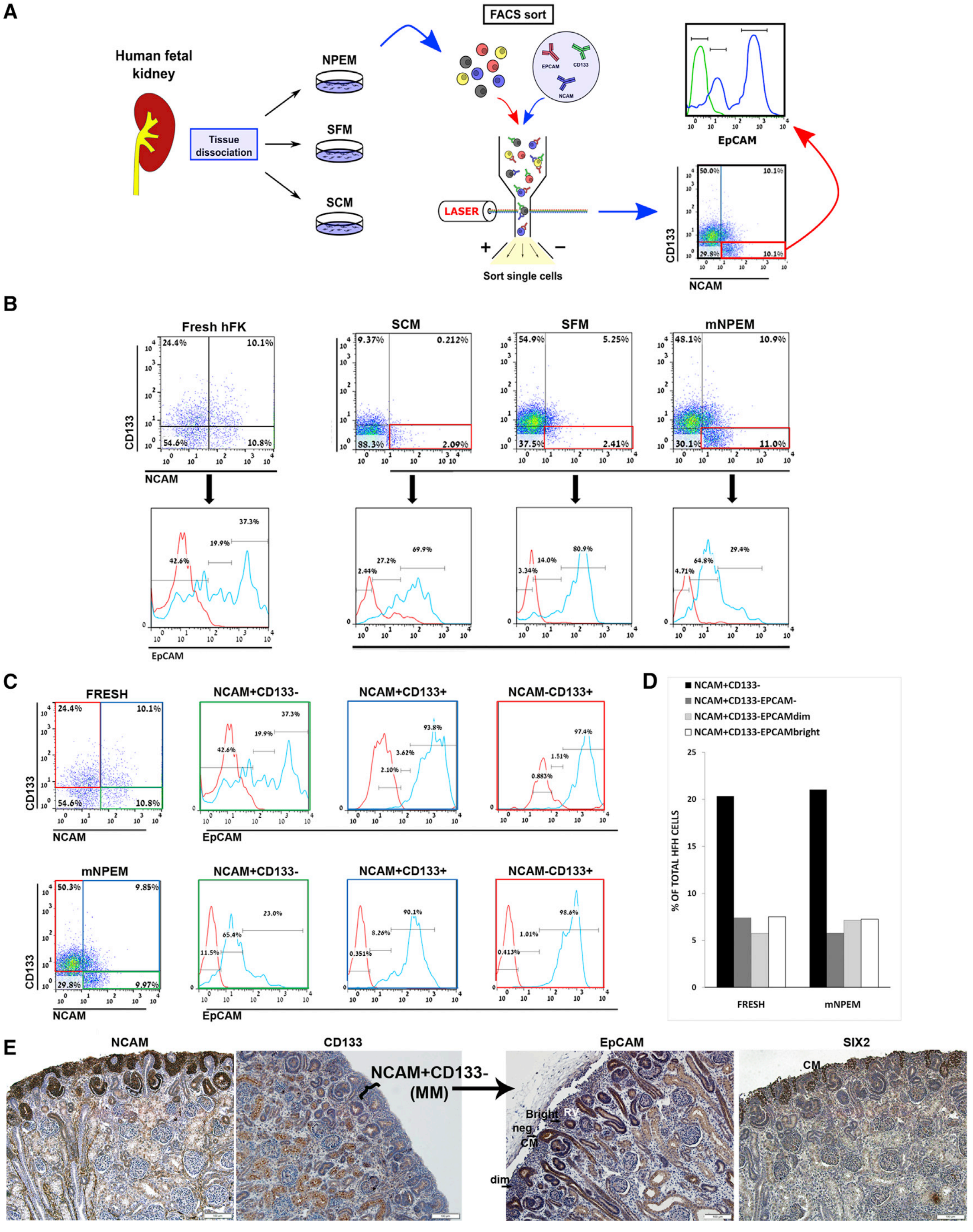

Figure 2. NCAM⁺CD133⁻ Cells Grown in mNPEM Contain Early Nephrogenic Lineages that Can Be Dissected according to EpCAM Expression

Mid-gestation hFKs were dissociated and cultured in serum-containing medium (SCM), serum-free medium (SFM), and mNPEM. FACS analysis was performed for NCAM1, a marker that was previously shown to enrich for the CM and early epithelial structures, CD133 (PROM1), a marker that was previously shown to enrich for differentiating and mature epithelial nephron tubules in the fetal kidney, and EpCAM, an epithelial marker (Pode-Shakked et al., 2016; Shapiro et al., 2011).

(A) Schematic representation of experiments.

(legend continued on next page)

nephron progenitor expansion medium (mNPEM) (Brown et al., 2015), we initially cultured hFK in each medium for three passages (Figure 1B). Cells grown in SFM or SCM for this time period displayed mostly cobblestone (a characteristic feature of epithelial cells) and spindle-shaped (a characteristic feature of mesenchymal cells) morphology, respectively (Figure 1B, left). In contrast, cells grown on MG (Matrigel matrix)-coated plates in mNPEM displayed morphological heterogeneity showing cobblestone, spindle-shaped, and ovoid-like cells and were organized in unique "niches." Each niche included a center of small ovoid-like crowded cells surrounded by larger cobblestone cells and crowned by an outer circle of spindle-shaped cells (Figure 1B). We next stained these cell cultures for SIX2 (as a marker for the nephron progenitors) and EpCAM (epithelial cell adhesion molecule) (as a marker for renal epithelial differentiation). As speculated, in hFK cultures grown in mNPEM, the small round cells that localize in the center of each niche stain for SIX2, while the cells in the periphery stain for EpCAM. In contrast, cells grown in either SFM or SCM for 7 days showed no SIX2 expression and were extensively stained for EpCAM (Figure 1B). Thus, only hFK cells cultured in mNPEM display phenotypic diversity forming distinct niches following even three passages in vitro.

To examine whether mNPEM actually preserves the diverse nephron epithelial cell lineages of fresh hFK, including CM, we used microfluidic multiplex qPCR (Biomark, Fluidigm) on 48 selected genes (Table S1) to compare the gene expression of hFK cells grown in each of the aforementioned growth media and freshly dissociated hFK cells (Figure 1C). Bulk gene expression of mNPEM-cultured hFK cells revealed a dominant CM identity (NCAM$^+$SIX2$^+$) and reduction of stromal elements (FOXD1$^+$) compared with other culture media and fresh tissue (see also Figure S1). It can also be seen that mNPEM best preserves markers for both mesenchymal (CITED1, OSR1, PAX2, SALL1, SNAI1, SNAI2, WT1) and epithelial (CDH1, CDH6, EPCAM) nephric lineages.

Finally, at the protein expression level, immunofluorescence staining of hFK cells grown in mNPEM for 14 days for SIX2 and EpCAM showed preservation of the aforementioned niches containing small SIX2$^+$ cells and surrounded by EpCAM$^+$ cells (Figures 1D and S2). Accordingly, some of the cells in the margins of each niche showed double staining for both SIX2 and EpCAM, suggesting a transitional state of differentiation (Figure 1D, upper panel). Other areas in the same culture showed pure epithelial colonies marked by EpCAM alone without SIX2 expression (Figure 1D, lower panel). Finally, quantification of SIX2-expressing cells in the mNPEM cultures (at 7 and 14 days) showed preservation of SIX2 percentages in culture (11% and 13%, respectively) (Figure 1E).

Thus, hFK kidney cells grown as monolayers in mNPEM maintain cellular diversity similar to that of fresh uncultured cells, including preservation of MET elements of nephrogenesis and reduction of interstitial cells.

NCAM$^+$CD133$^-$ Cells Grown in mNPEM Contain Early Nephrogenic Lineages that Can Be Dissected According to EpCAM Expression

hFK cells were grown in mNPEM (Brown et al., 2015) for 7 days, after which they were analyzed for the presence of three surface markers: NCAM1 (NCAM), previously shown to enrich for the CM and early epithelial structures (Pode-Shakked et al., 2016); CD133 (PROM1), previously shown to enrich for differentiating and mature nephron tubular epithelia in the fetal kidney; and EpCAM, an epithelial differentiation marker (Figure 2A). FACS analysis of hFK cultured in mNPEM was compared with cells grown in SCM or SFM for 7 days (Figure 2B). Dissection of the NCAM$^+$CD133$^-$ cell fraction, particularly in mNPEM, revealed three early

(B) Representative FACS analysis of fresh hFK and hFK cultured in either mNPEM, SFM, or SCM for 7 days. Cells that were grown in mNPEM preserve the NCAM1$^+$CD133$^-$ cell fraction, similar to freshly dissociated cells (11% and 10.8%, respectively). In contrast, SCM and SFM show decreased percentages of NCAM1$^+$CD133$^-$ cells (2.09% and 2.41%, respectively). Moreover, in both freshly dissociated hFK and cells grown in mNPEM, most cells are EpCAM^{dim} or EpCAM$^-$ (42.6 + 19.9 = 62.5% and 4.71 + 64.8 = 69.51%, respectively), while cells grown in SCM or SFM show a much smaller percentage of EpCAM^{dim} or EpCAM$^-$ populations (2.44 + 27.2 = 29.64% and 3.34 + 14 = 17.34%, respectively).

(C) Close examination of the different cell subpopulations in fresh hFK and mNPEM cultures shows that in addition to maintaining the NCAM$^+$CD133$^-$ subpopulation and EpCAM distribution, the NCAM1$^+$CD133$^+$ and NCAM$^-$CD133$^+$ that represent the more epithelial differentiating and differentiated cells are also preserved in these cultures in a way that is comparable with fresh cells (note that the mNPEM cells here are from a different biological replicate than those in B, but the numbers are comparable). The gradual increase in EpCAM^{bright} cells (to ~98% and 97% in the NCAM$^-$/CD133$^+$ cell population) in both mNPEM-grown and fresh hFK cells is consistent with earlier findings and further stresses the preservation of all hFK epithelial lineages in mNPEM. See also Figure S2.

(D) Bar graph showing mean percentages of NCAM$^+$CD133$^-$ cell subpopulations in fresh hFK and mNPEM cultures and the distribution of EpCAM subpopulations within this fraction (data were averaged over three independent experiments from three different hFK sources, n = 3).

(E) Immunohistochemical staining of hFK tissue (from a 22-week embryo) for NCAM, CD133, EpCAM, and SIX2 showing that within the NCAM$^+$CD133$^-$ section of the MM, the EpCAM^{bright} cells correspond to early epithelial differentiation (renal vesicles in particular), while the EpCAM$^-$ and EpCAM^{dim} cells are restricted to the most cortical region containing the SIX2$^+$ cells of the CM and early CM progeny. MM, metanephric mesenchyme; CM, condensed mesenchyme; RV, renal vesicle; T, tubules. Scale bars, 100 μm. See also Figure S2 and Table S2.

renal subpopulations ($NCAM^+CD133^-EpCAM^-$, $NCAM^+CD133^-EpCAM^{dim}$, and $NCAM^+CD133^-EpCAM^{bright}$) putatively spanning from the CM to the renal vesicle stage, respectively. hFK grown in mNPEM exclusively showed preservation of the $NCAM^+CD133^-$ subpopulation, similar to freshly dissociated hFK cells (~10%–11%, Figures 2B and 2C). On the contrary, the $NCAM^+CD133^-$ subpopulation was significantly reduced in cells grown in SFM/SCM for 7 days (2%–3%, Figure 2B; see also Figure S2 and Table S2). Moreover, the $NCAM^+CD133^-EpCAM^{-/dim}$ fraction, putatively representing early nephrogenesis ($SIX2^+$ cells), was preserved only in mNPEM, similar to fresh hFK cells (~60%–70%). On the contrary, in SFM and SCM cultures the $NCAM^+CD133^-$ cells were mostly $EpCAM^{bright}$ (~70%–80%), while $EpCAM^{dim}$ or $EpCAM^-$ were minimally preserved (Figure 2B; see also Figure S2 and Table S2). Alongside the preservation of early nephrogenesis in mNPEM, later epithelial renal lineages were also maintained in mNPEM compared with freshly dissociated hFK. $NCAM^+CD133^-EpCAM^{bright}$ (putative early nephron differentiation in late transition from mesenchyme to epithelia), $NCAM^+CD133^+$ (corresponding to the S-/comma-shaped bodies), and $NCAM^-CD133^+$ (corresponding to differentiated nephron epithelia) subpopulations were all preserved (Figures 2C, 2D, and S2; Table S2). Table S2 summarizes the FACS data of NCAM, CD133, and EpCAM in fresh and cultured hFK from three different sources.

To demonstrate the in situ distribution of EpCAM in the $NCAM^+CD133^-$hFK compartment, we immunostained hFK tissue for NCAM, CD133, SIX2, and EpCAM (Figure 2E). It can be seen that within the $NCAM^+CD133^-$ (MM) section, EpCAM is absent from the CM. Early CM progeny maintains SIX2 and starts to express EpCAM in low levels ($EpCAM^{dim}$). In the renal vesicles within the $NCAM^+CD133^-$ section, EpCAM is upregulated ($EpCAM^{bright}$) (Figure 2E, third panel from left). Thus, the most cortical segment containing $SIX2^+$ cells seems to overlap with the $NCAM^+CD133^-EpCAM^{dim/neg}$ cell subpopulation. We could also discern later stages of renal MET, in particular, S-/comma-shaped bodies ($NCAM^+CD133^+EpCAM^{bright}$) and differentiated tubules ($NCAM^-CD133^+EpCAM^{bright}$).

Thus, mNPEM allows preservation of early renal nephrogenesis while maintaining differentiated epithelial lineage.

Single-Cell Gene Expression Analysis of Prospectively Isolated hFK-Cultured Cells Reveals Representation of Major Stages of Human Nephrogenesis

To further characterize the mNPEM-cultured hFK cells at the single-cell level, we performed microfluidic single-cell qPCR. hFK cells cultured in mNPEM for 7 days were FACS sorted and ~80 single cells from three fractions ($NCAM^+CD133^-EpCAM^-$, $NCAM^+CD133^-EpCAM^{dim}$, and $NCAM^+CD133^+$) were collected into 96-well plates. For each individual cell, we measured the expression of 48 gene targets known to be involved in kidney development and tumorigenesis (Little et al., 2010; Harding et al., 2011; Table S3; Figure 3A). We identified four major cell subpopulations representing distinct transcriptional states that could be related to the major cell types participating in kidney development (Figure 3B).

The $NCAM^+CD133^-EpCAM^-$ fraction enriched for two mesenchymal cell subpopulations: a CM population, overexpressing the genes SIX2, WT1, OSR1, CITED1, EYA1, SIX1, and CDH11, and a second mesenchymal subpopulation overexpressing the genes SERPINE1, ZEB1, and CDH11 (but not the CM markers SIX1, SIX2, EYA1, and CITED1, or the epithelial markers CDH1, KRT19, and EpCAM). To identify this population, we searched the microarray data in the GUDMAP database (http://www.gudmap.org) for a cell population in the mouse embryonic kidney having similar characteristics.

We found that the nephrogenic interstitial cells in embryonic day 15 (E15.5) mouse embryos overexpress the gene SERPINE2, a paralog of SERPINE1, together with ZEB1 and CDH11 (Figure S3). In this same cell fraction the CM markers SIX1, SIX2, EYA1, and CITED1, and the epithelial markers CDH1, KRT19, and EpCAM were underexpressed. Therefore, we hypothesize that the second non-CM cell subpopulation (in the human cells) represents the nephrogenic interstitium, including the uninduced MM/stromal cells of the nephrogenic zone.

The $NCAM^+CD133^-EPCAM^{dim}$ fraction was found to enrich for a MET state. Many of these cells overexpressed some CM-characteristic genes (SIX2, WT1, CDH11) but also genes characteristic of the early nephron epithelium (CDH6, KRT18, and KRT19), as well as PAX2, which is a marker for mesenchyme-derived epithelium (Cho et al., 1998).

The $NCAM^+CD133^+$ fraction is enriched for cells overexpressing genes characteristic of early nephric epithelial cells: EPCAM, CDH1, CDH6, PAX2, KRT18, and KRT19. Within this population we observed cells with proximal ($CDH6^{high}/WT1^{high}$) and distal ($CDH1^{high}$) tubular phenotypes. Furthermore, we also observed expression of ALDH1A1 and ALDH1A2, genes that were previously shown to be localized to more differentiated tubular elements in the human fetal kidney (Pode-Shakked et al., 2013).

Wilms' tumor was previously shown to enrich for early developmental cell types resembling the fetal CM (Pode-Shakked et al., 2013). We therefore performed the same single-cell analysis on Wilms' tumor for comparison with the single-cell gene expression profiling in mNPEM-cultured hFK cells. We have previously shown using xenograft transplantation experiments that the $NCAM^+/ALDH1^+$ cell

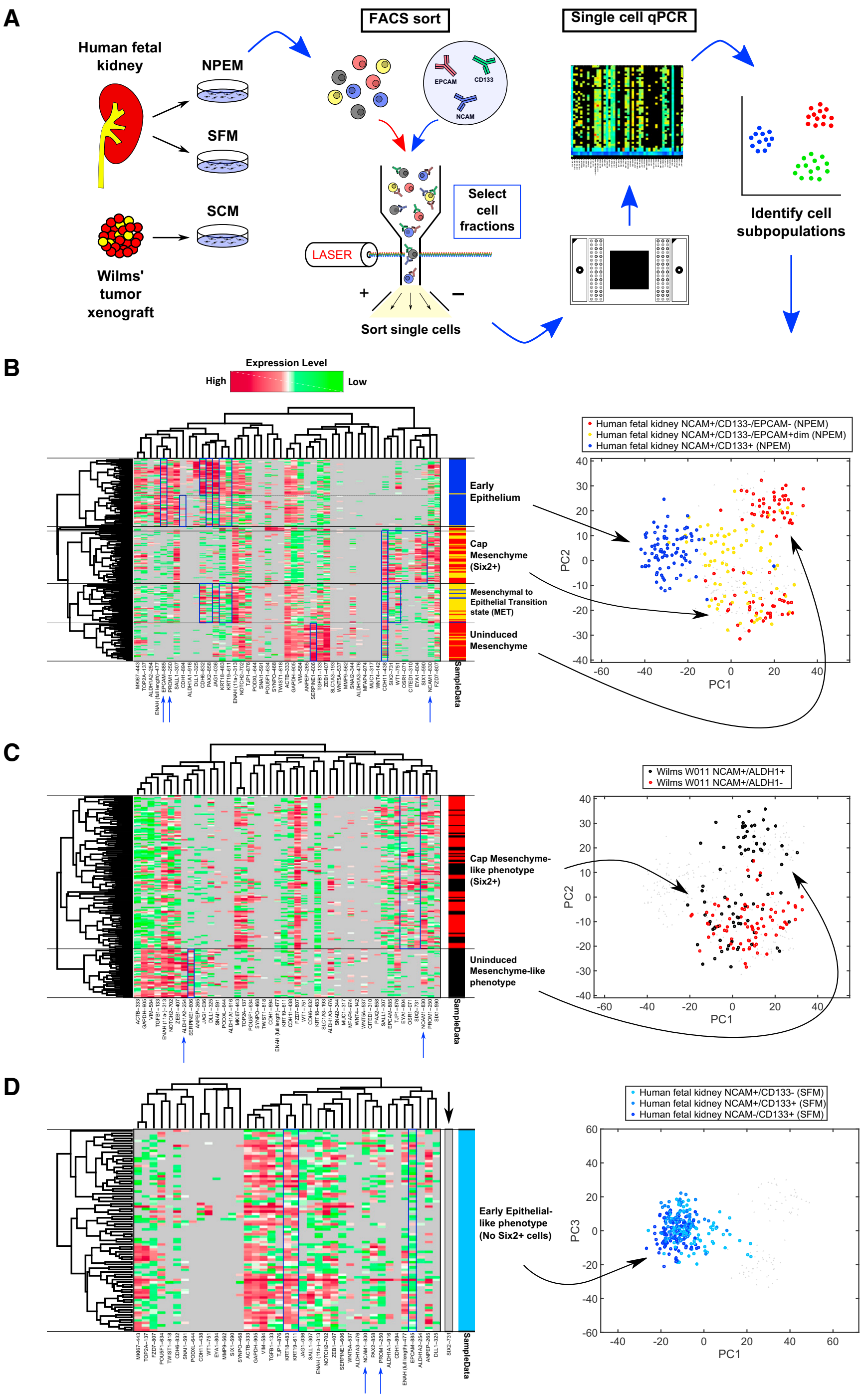

Figure 3. Single-Cell Gene Expression Analysis of Prospectively Isolated hFK-Cultured Cells Reveals Cell Subpopulations Representing All Stages of Human Nephrogenesis

(A) Schematic representation of the experiments performed.

(B) Gene expression heatmap and corresponding PCA plot of ~240 single cells (rows) and 47 genes (columns) measured simultaneously from each cell. Gene expression levels (in terms of threshold cycles, Ct) were standardized and clustered such that phenotypically similar cells are grouped next to each other (red, high expression; green, low expression; gray, no expression). Cells were isolated from human fetal kidney, cultured in mNPEM for 7 days, and sorted by FACS into three fractions: NCAM$^+$CD133$^-$ EPCAM$^-$, NCAM$^+$CD133$^-$EPCAM^{dim}, and NCAM$^+$ CD133$^+$. The colors in the column on the right of the heatmap and in the PCA plot represent the FACS-sorted fraction of origin of each individual cell. We identified distinct cell subpopulations whose expression profiles correspond to the CM (SIX2$^+$WT1$^+$OSR1$^+$ CITED1$^+$EYA1$^+$SIX1$^+$CDH11$^+$), early nephric epithelia (EPCAM$^+$CDH1$^+$CDH6$^+$PAX2$^+$KRT18$^+$ KRT19$^+$), and a MET-transition state (many of which cells express both CM and epithelial markers). An additional mesenchymal subpopulation (SERPINE1$^+$ZEB1^{high}CDH11$^+$ SIX2$^-$) is hypothesized to correspond to the nephrogenic interstitium (containing the uninduced metanephric mesenchymal/stromal cells of the nephrogenic zone).

(C) Similar analysis of ~160 single cells from the tumorigenic (NCAM$^+$ALDH1$^+$) and non-tumorigenic (NCAM$^+$ALDH1$^-$) cell fractions of a late (passage 10) blastemal Wilms' tumor-PDX reveals two cell subpopulations: a CM-like subpopulation (EYA1$^+$OSR1$^+$SIX2$^+$ SIX1$^+$), found in both cell fractions, and an uninduced mesenchyme-like subpopulation (SERPINE1$^+$SIX2$^-$) found only in the tumorigenic fraction.

(D) All three cell fractions (NCAM$^+$CD133$^-$, NCAM$^+$CD133$^+$, and NCAM$^-$CD133$^+$) sorted from hFK cells cultured in SFM for 10 days have a nephric epithelial phenotype (EPCAM$^+$KRT18$^+$KRT19$^+$SIX2$^-$). In the NCAM$^+$CD133$^-$ fraction, we did find a small number of more early mesenchymal cells (CDH11$^+$), but no measurable SIX2$^+$ cells were found within the ~240 cells that we measured. In all heatmaps, genes that had zero expression in all cells are not shown (apart from SIX2).

Note that PCA analysis in (B) and (C) was performed on a union of all cell fractions from hFK-mNPEM and Wilms' tumor cells, whereas in (D) only hFK-SFM cell fractions were included, along with the hFK-mNPEM mesenchymal fraction (NCAM$^+$CD133$^-$EPCAM$^-$) to allow for better visual comparison. See also Figure S3; Tables S1 and S3.

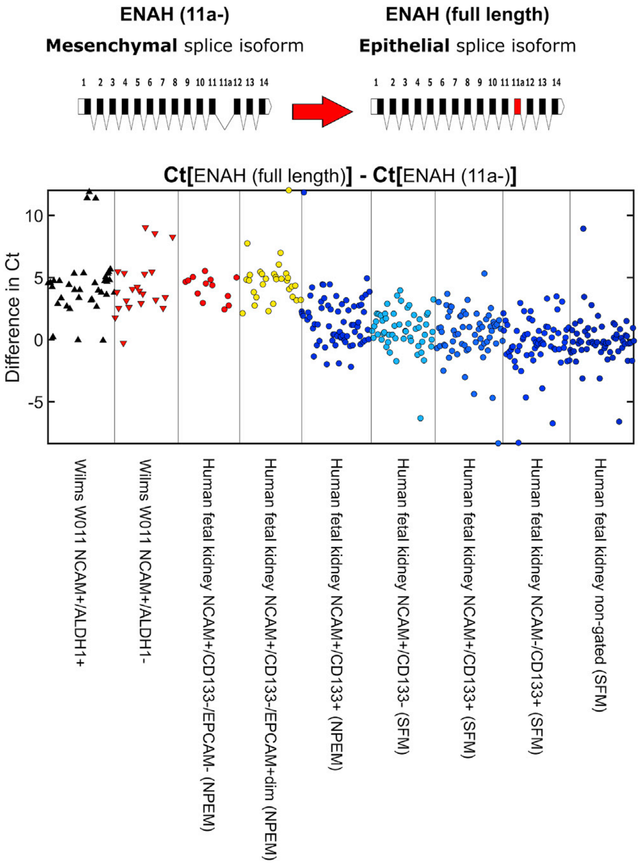

Figure 4. Splice Isoform Expression Analysis at the Single-Cell Level Shows Epithelial and Mesenchymal Phenotypes in Prospectively Isolated hFK-Cultured Cells and WT-PDX

Shown is the relative expression (in terms of the difference in qPCR threshold cycles, Ct) between the epithelial and mesenchymal isoforms of the gene ENAH. For clarity, only cells in which both isoforms were detected are shown. It can be seen that hFK cells cultured in mNPEM contain subpopulations with both a predominant mesenchymal isoform (NCAM$^+$CD133$^-$EPCAM$^-$ and NCAM$^+$CD133$^-$EPCAM^{dim}) and a predominant epithelial isoform (NCAM$^+$CD133$^+$). Both fractions (NCAM1$^+$ADLH1$^+$ and NCAM1$^+$ ADLH1$^-$) of the blastemal Wilms' tumor-PDX had strong overexpression of the mesenchymal isoform, and all three fractions (NCAM$^+$CD133$^-$, NCAM$^+$CD133$^+$, and NCAM$^-$CD133$^+$) of hFK cells cultured in SFM overexpressed the epithelial isoform.

fraction from within the Wilms' tumor blastema is enriched for cancer stem cells (CSCs) (Pode-Shakked et al., 2013). Therefore, we used flow cytometry to sort two cell fractions from a late (passage 10) blastemal Wilms' tumor patient-derived xenograft (WT-PDX): NCAM$^+$/ALDH1$^+$ and NCAM$^+$/ALDH1$^-$. We found (Figure 3C) that while both cell fractions contain a significant CM-like subpopulation (overexpressing the genes EYA1, OSR1, SIX2, and SIX1), only the tumorigenic NCAM$^+$/ALDH1$^+$ fraction contains a cell subpopulation resembling the nephrogenic interstitium/uninduced mesenchyme (overexpressing SERPINE1 and almost not expressing SIX2). This indicates that the uninduced mesenchyme-like population is essential for tumorigenicity. Additionally, consistent with the overall blastemal characteristics of the WT-PDX, single-cell resolution disclosed only a repertoire of mesenchymal cells, and there was no measurable population of nephric epithelial cells (Figure 3C).

We have previously shown that upon short-term culture (7 days) in SFM, hFK NCAM$^+$CD133$^-$ sorted cells, albeit representing a diminutive cell fraction (~0.5%), maintain some CM phenotype (SIX2 high) (Pode-Shakked et al., 2016; Shapiro et al., 2011). However, after culturing for 10 days in SFM we observed rapid differentiation into epithelia. Consistent with this observation, single-cell qPCR analysis for hFK cells that were grown in SFM for 10 days showed only the epithelial-like phenotype (overexpressing EPCAM, KRT18, and KRT19) and did not express measurable SIX2 at all, as well as other markers of early nephrogenesis (Figure 3D). These cells thus represent an early epithelialization stage likely equivalent to NCAM$^+$CD133$^-$EpCAM^{bright} cell fraction in the mNPEM.

To summarize, only fetal human cells grown in mNPEM capture the full repertoire of cell subpopulations that are found in the early developing fetal kidney.

Splice Isoform Expression Analysis at the Single-Cell Level Shows that hFK Cells Cultured in mNPEM Contain Both Epithelial and Mesenchymal Phenotypes, thus Recapitulating the MET in Human Nephrogenesis

We previously showed, using "bulk" RNA sequencing measurements of human fetal cells cultured in SFM, that during the MET in early kidney development many genes undergo splice isoform switching similar to that found in epithelial to mesenchymal transition in breast cancer (Pode-Shakked et al., 2013). One such gene is ENAH (hMena), which was found to switch between two isoforms: a mesenchymal isoform lacking exon 11a, and an epithelial isoform including it (Di Modugno et al., 2012; Shapiro et al., 2011; Pignatelli et al., 2014). We next demonstrated this at the single-cell level (Figure 4) by choosing primers specific to the ENAH (11a-) and ENAH (full-length) isoforms and measuring their levels using single-cell qPCR.

We found that fetal human cells grown in mNPEM contain both subpopulations with a predominant mesenchymal isoform (NCAM$^+$CD133$^-$EpCAM$^-$ and NCAM$^+$ CD133$^-$EpCAM^{dim}) and a predominant epithelial isoform (NCAM$^+$CD133$^+$). Both fractions of Wilms' tumor (NCAM1$^+$ ADLH1$^+$ and NCAM1$^+$ADLH1$^-$) showed a strong overexpression of the mesenchymal isoform, and all fractions of the fetal cells grown in SFM (NCAM$^+$CD133$^-$, NCAM$^+$CD133$^+$, and NCAM$^-$CD133$^+$) expressed the

epithelial isoform. Therefore, only human fetal cells grown in mNPEM were found to capture the MET at both gene expression and splice isoform levels.

DISCUSSION

Herein, by utilizing a nephron progenitor-preserving medium and a combination of surface markers that allow for cell sorting and single-cell analysis of sorted fractions, we map single hFK cells from expanded cultures and show for the first time the preservation of a spectrum of cell types in vitro that constitute human nephrogenesis in vivo. Once ex vivo maintenance of the various cell types participating in nephrogenesis, including stem/progenitor cells, differentiating, and differentiated renal lineages was achieved, deeper phenotyping at single-cell resolution could be carried out.

Culturing hFK in mNPEM instantly showed morphological heterogeneity, including specific undifferentiated stem cell niches and differentiating epithelial cells, which could be further exploited to show an EpCAM expression gradient (EpCAM^{-}, EpCAM^{dim}, EpCAM^{bright}) within the NCAM1^{+}CD133^{-} hFK cell fraction that was previously suggested to enrich for nephron progenitors (Metsuyanim et al., 2009; Pode-Shakked et al., 2016). This gradient, and especially the NCAM1^{+}CD133^{-}EpCAM^{-} hFK cells (a presumed early fraction), were not observed when hFK cells were grown and passaged in SFM or SCM. Since we are dealing with human biological samples, antigenic expression may differ between hFKs with resultant variable percentage of NCAM^{+}CD133^{-} cells. Nevertheless, this variability does not affect the distribution of the different compartments according to EpCAM, which are constantly preserved.

The appearance of discrete cell subpopulations when growing hFK in mNPEM afforded the opportunity to measure gene expression at single-cell resolution in sorted cell fractions and to decipher the respective stages of human renal development. Reassurance that cells of early nephrogenesis are indeed preserved in culture came for microfluidic multiplexed qPCR experiments that analyzed bulk RNA from hFK cells grown in mNPEM, SCM, and SFM, and demonstrated differential gene expression compatible with preservation of the CM in the hFK cells grown in mNPEM. This was further verified by widespread protein expression of SIX2 in hFK-mNPEM culture at P3, as well as preservation of epithelial nephric lineages.

Single-cell gene expression analysis of cell subpopulations sorted from hFK expanded in mNPEM using the Biomark system (Dalerba et al., 2011) provided a more detailed picture that better characterized the cell types expanded in culture, and pinpointed for the first time the in vitro preservation of early human nephrogenesis. Accordingly, two populations of uninduced and induced mesenchyme (CM) are expanded in the NCAM1^{+}CD133^{-}EpCAM^{-} hFK cell fraction. An additional type of cells expanded in mNPEM and residing within the NCAM1^{+}CD133^{-} fraction appear to be in transition from mesenchyme toward epithelia (NCAM1^{+}CD133^{-}EpCAM^{dim} cells). While, as expected, SIX2 expression can be traced to NCAM^{+} CD133^{-}EPCAM^{-} cells, there appears to be residual SIX2 expression also in the NCAM^{+}CD133^{-}EPCAM^{dim} fraction, defining a unique early CM-derived cell type expressing both a stem and differentiating phenotype. This argues against a model whereby SIX2 is shut down and only after which epithelialization starts to emerge, but rather informs about the presence of an intermediate phenotype. In this regard, immunostaining showed that NCAM^{+}CD133^{-} EPCAM^{bright} cells mark renal vesicles from which SIX2 is completely absent. Importantly, while mNPEM favors expansion of cell types of early nephrogenesis, we were also able to observe differentiated nephron epithelia in later stages (NCAM1^{+}CD133^{+}) at single-cell resolution. In contrast, we found no evidence for expansion of non-nephrogenic mature lineages such as pericytes or endothelium.

To fully appreciate and to validate the cell repertoire expanded in mNPEM (see principal component analysis [PCA] plots in Figure 3), we added additional human samples in which this repertoire appears only in part; for instance, in blastema-enriched human Wilms' tumor-PDX, single-cell analysis and PCA plots disclose the early uninduced and induced mesenchymal cell types but show total absence of the epithelial component. In contrast, in hFK expanded in serum-free media to prolonged time points (10 days), single-cell analysis and PCA plots show disappearance of the mesenchymal fractions. The diversity of the mNPEM-cultured hFK cells was further demonstrated by measuring mesenchymal and epithelial associated splice isoforms of the gene ENAH at the single-cell level (Figure 4). These measurements showed that only mNPEM-cultured hFK cells contain subpopulations with either a predominant mesenchymal isoform (NCAM^{+}CD133^{-}EPCAM^{-} and NCAM^{+}CD133^{-}EPCAM^{dim}) or a predominant epithelial isoform (NCAM^{+}CD133^{+}). In comparison, both cell fractions of the Wilms' tumor xenograft overexpressed the mesenchymal isoform and all three fractions of the SFM-cultured hFK cells expressed the epithelial isoform.

Recently, directed differentiation of pluripotent stem cells to an organoid that approximates a 10-week human fetal kidney has been shown (Dekel, 2016; Takasato et al., 2015; Morizane et al., 2015; Tanigawa et al., 2016). It would be interesting to dissect, at the single-cell level,

the cellular repertoires expanded in the organoid and compare it with single-cell heatmaps we have generated for expanded hFK. We acknowledge that the Biomark system allowing 48 gene measurements in a cell and not the full transcriptome may be limited. Nevertheless, the discrete hFK cell types unraveled by this system may already provide a screen for any attempt to analyze the outcome of induced pluripotent stem cell to hFK differentiation. Moreover, since a full repertoire of developing human kidney cells is expanded in the mNPEM hFK cultures, we propose them as ideal for modeling gene defects that hamper nephron differentiation at varying stages of the MET axis (Vivante et al., 2013), drug screens that aim to boost expansion of human nephron progenitors, and toxicological screens that aim to determine harmful effects at the stem cell level or more differentiated cell progeny.

EXPERIMENTAL PROCEDURES

Ethics Statement

This study was conducted according to the principles expressed in the Declaration of Helsinki and was approved by the Institutional Review Boards of Sheba, Hadassah-Ein Kerem, and Asaf Harofeh Medical Centers.

Human Fetal Kidney Samples

hFK were collected from elective abortions. Fetal gestational age ranged from 15 to 22 weeks. All studies were approved by the local ethical committee and informed consent was given by the legal guardians of the patients involved according to the Declaration of Helsinki.

Establishment of Single-Cell Suspension from Human Fetal Kidney

Each tissue was processed for the formation of a single-cell suspension as previously described (Pode-Shakked et al., 2013). In brief, collected tissues were washed with cold Hank's balanced salt solution (Invitrogen) and minced into ~1-mm cubes using sterile surgical scalpels. The dissected tissue was then incubated for 2 hr at 37°C with Iscove's modified Dulbecco's medium (IMDM) (Invitrogen) supplemented with 0.1% collagenase IV (Invitrogen). The digested tissue was then gradually forced through a 100-μm cell strainer to achieve a single-cell suspension.

Primary hFK Cell Cultures

Primary hFK cell cultures were performed as previously described (Harari-Steinberg et al., 2013; Metsuyanim et al., 2009; Pode-Shakked et al., 2013). Single-cell suspensions of hFK were resuspended in a growth medium (SCM, SFM, or mNPEM) and plated in flasks. SCM was composed of IMDM (Biological Industries) supplemented with 10% fetal bovine serum (Invitrogen), 1% Penicillin-streptomycin (pen-strep) 100 M, 1% L-glutamine (both from Biological Industries), 100 ng/mL epidermal growth factor (EGF), 100 ng/mL basic fibroblast growth factor (FGF), and 10 ng/mL stem cell factor (all growth factors purchased from Peprotech Asia). For passaging, cells were detached using 0.05% trypsin/EDTA (Invitrogen). SFM was composed of N2 medium (Biological Industries) supplemented with 1% pen-strep 100 M, 1% L-glutamine, 0.4% B27 supplement (Gibco), 4 μg/mL heparin sodium (Intramed), 1% non-essential amino acids, 1% sodium pyruvate, 0.2% CD lipid concentrate (all from Invitrogen), 2.4 mg/mL glucose, 0.4 mg/mL transferrin, 10 mg/mL insulin, 38.66 μg/mL putrescine, 0.04% sodium selenite, 12.6 μg/mL progesterone (all from Sigma-Aldrich), 10 ng/mL FGF, and 20 ng/mL EGF. For passaging, cells were detached using a non-enzymatic cell dissociation solution (Sigma-Aldrich). Cells were incubated as described previously (Pode-Shakked et al., 2009). All assays were conducted with low-passage cultured cells. For mNPEM, cells were plated onto 96-well plates pre-coated with Matrigel (BD Biosciences) and cultured in APEL medium (Stem Cell Technologies) supplemented with 10 μM Y-27632, 1.25 μM CHIR99021 (Stemgent), 200 ng/mL FGF9 (R&D Systems), 30 ng/mL bone morphogenetic protein 7 (BMP7) (Sigma-Aldrich), 125 nMLDN-193189 (R&D Systems), and 1 mg/mL heparin (Sigma-Aldrich). Insulin-like growth factor 1 (IGF1), IGF2, and BMP4 were excluded from the original medium (Brown et al., 2015) due to lack of effect on the cultured cells probably since they are unsuitable for human cells. For passaging, cells were dissociated by incubation with TrypLE for 2 min at 37°C. mNPEM was changed every 2 days. The cells were observed using Nikon Eclipse TS100 and Nikon Digital Sight cameras.

Fluorescence-Activated Cell Sorting Analysis

FACS analyses were performed on cells originating from three independent samples of hFK (ranging from 15 to 22 weeks of human gestation: hFK103, hFK104, and hFK105) as previously described (Dekel et al., 2006b). Cells grown in either medium (mNPEM, SFM, SCM) were harvested using 0.05% trypsin/EDTA (Gibco) or a non-enzymatic cell dissociation solution (Sigma-Aldrich), and the number of viable cells was determined using Trypan blue staining (Invitrogen). Cells (1×10^5 in each reaction) were suspended in 50 μL of FACS buffer (0.5% BSA and 0.02% sodium azide in PBS [Sigma-Aldrich and Invitrogen, respectively]) and blocked with FcR Blocking Reagent (Miltenyi Biotec) and human serum (1:1) for 15 min at 4°C. Surface antigens were labeled by incubation with fluorochrome-conjugated mouse anti-human CD133/1-Vio-bright (fluorescein isothiocyanate equivalent) (Miltenyi Biotech, catalog #130-105-226), mouse anti-human NCAM-allophecoaritin (eBioscience, #17-0569-42), and anti-EpCAM (CD326)-phecoaritin (eBioscience, #12-9326-42) for 45 min in the dark at 4°C to prevent internalization of antibodies. 7-Amino-actinomycin-D (7AAD; eBioscience, #00-6993-50) was used to select for viable cells. All washing steps were performed in FACS buffer. Quantitative measurements were made from the crosspoint of the immunoglobulin G isotype graph with the specific antibody graph. Data were additionally analyzed and presented using FlowJo software.

FACS Sorting

Cells were harvested as described above, filtered through a 30-μm nylon mesh before final centrifugation, and resuspended in FACS

buffer. A FACSAria III sorter was used to enrich for cells expressing surface markers. An 85-μm nozzle (BD Biosciences), sheath pressure of 20–25 pounds per square inch, and an acquisition rate of 1,000–3,000 events per second were used. Single viable cells were gated on the basis of 7AAD and then physically sorted into 96-well plates for single-cell gene expression analysis.

Bulk RNA Purification

Bulk total RNA was prepared from $\sim 1.5 \times 10^5$ cells using the Direct-zol RNA MiniPrep kit (Zymo Research) according to the manufacturer's instructions and stored in −80°C.

Microfluidic Single-Cell qPCR

Single cells were sorted by FACS into individual wells of 96-well plates. After cell lysis, mRNA levels were measured by microfluidic single-cell qPCR using the Biomark system (Fluidigm) according to the manufacturer's instructions. This resulted in 48 gene expression values (measured in threshold cycles, Ct) for each one of the cells sorted. We analyzed approximately 80 cells from each cell fraction. qPCR standard curves were created using serial dilutions of "bulk" RNA containing a mixture of HeLa total RNA (Thermo Fisher Scientific) and RNA from adult and fetal human kidneys. TaqMan gene expression primers and probes were purchased from Thermo Fisher Scientific.

For clustering analysis, we standardized the expression levels of each gene individually by subtracting the mean and dividing by 3-times the SD of expressing cells. Then all values were truncated into the range [−1, +1] as previously described (Dalerba et al., 2011). Clustering was performed using complete linkage and correlation distance (MATLAB).

Microfluidic multiplex qPCR from "bulk" total RNA samples was done similarly; however rather than sorting single cells into 96-well plates, 1 μL of total RNA from each sample was inserted into each well. Prior to standardization and clustering analysis, qPCR threshold cycles (Ct) were normalized to the gene ACTB. Similar results were obtained by normalizing to GAPDH. See also Supplemental Experimental Procedures.

Agar Cyto Cell Block Preparation and Immunofluorescence Staining

A total of 10^6 cells were fixed in 4% paraformaldehyde (PFA) for 2 hr, washed in PBS, and suspended in low-melting-point agar. After becoming solid at 40°C the agar block was transferred into PFA overnight at 40°C, washed with distilled water, and embedded. Five-micrometer sections of paraffin-embedded agar blocks were mounted on super frost-plus glass and incubated at 60°C overnight. Following deparaffinization and antigen retrieval with Zytomed Systems OmniPrep Solution, slides were incubated in Cas-Block solution for 1 hr at room temperature. The primary antibodies SIX2 (11562-1-AP, Proteintech) and Ki67 (VP-K452, Vector Laboratories) were diluted in commercial antibody diluent, incubated for 1 hr at room temperature, washed with PBS-Tween (0.05%), and incubated with secondary antibodies for 1 hr at room temperature. Following PBS-Tween washes, slides were mounted with DAPI-containing mounting and covered with coverslips. Staining was evaluated with an Olympus BX51TF microscope and Scion color and monochrome digital cameras.

For experimental procedures for immunofluorescence staining of hFK cells, in vivo WT xenograft formation, single-cell gene expression analysis, and statistical analyses, see Supplemental Experimental Procedure.

SUPPLEMENTAL INFORMATION

Supplemental Information includes Supplemental Experimental Procedures, four figures, and three tables and can be found with this article online at http://dx.doi.org/10.1016/j.stemcr.2017.04.026.

AUTHOR CONTRIBUTIONS

B.D., N.P.-S., and T.K. designed the experiments. N.P-S., R.G., I.K., G.T., T.K., S.O., G.K., Y.G., and D.O. performed the experiments. B.D., N.P.-S., I.K., G.T., S.O., T.K., and O.H.-S. analyzed the data. B.D., N.P.-S., T.K., and R.G. wrote the manuscript.

ACKNOWLEDGMENTS

We wish to thank Jordan Kreidberg, Joseph V. Bonventre, Robert H. Singer, John S. Condeelis, Achia Urbach, Tali H. Bar-Lev, Nissim Ben-Haim, and all members of our labs for fruitful discussions and suggestions. This work was supported by the ICRF (grant nos. 15731 and 15450), the Israel Cancer Association (grant no. 20150916), and The Ziering Foundation (grant no. 45124) (B. D.). T.K., G.T., I.K., and S.O. are supported by the Israel Science Foundation (ICORE no. 1902/12 and grant nos. 1634/13 and 2017/13), the Israel Cancer Association (grant no. 20150911), the Israel Ministry of Health (grant no. 3-10146), and the EU-FP7 (Marie Curie International Reintegration grant no. 618592).

Received: September 6, 2016
Revised: April 24, 2017
Accepted: April 25, 2017
Published: May 25, 2017

REFERENCES

Boyle, S., Misfeldt, A., Chandler, K.J., Deal, K.K., Southard-Smith, E.M., Mortlock, D.P., Baldwin, H.S., and de Caestecker, M. (2008). Fate mapping using Cited1-CreERT2 mice demonstrates that the cap mesenchyme contains self-renewing progenitor cells and gives rise exclusively to nephronic epithelia. Dev. Biol. *313*, 234–245.

Brown, A.C., Muthukrishnan, S.D., and Oxburgh, L. (2015). A synthetic niche for nephron progenitor cells. Dev. Cell *34*, 229–241.

Cho, E.A., Patterson, L.T., Brookhiser, W.T., Mah, S., Kintner, C., and Dressler, G.R. (1998). Differential expression and function of cadherin-6 during renal epithelium development. Development *125*, 803–812.

Daar, A.S. (2006). The case for a regulated system of living kidney sales. Nat. Clin. Pract. Nephrol. *2*, 600–601.

Dalerba, P., Kalisky, T., Sahoo, D., Rajendran, P.S., Rothenberg, M.E., Leyrat, A.A., Sim, S., Okamoto, J., Johnston, D.M., Qian,

D., et al. (2011). Single-cell dissection of transcriptional heterogeneity in human colon tumors. Nat. Biotechnol. *29*, 1120–1127.

Dekel, B. (2016). The ever-expanding kidney repair shop. J. Am. Soc. Nephrol. *27*, 1579–1581.

Dekel, B., Burakova, T., Ben-Hur, H., Marcus, H., Oren, R., Laufer, J., and Reisner, Y. (1997). Engraftment of human kidney tissue in rat radiation chimera: II. Human fetal kidneys display reduced immunogenicity to adoptively transferred human peripheral blood mononuclear cells and exhibit rapid growth and development. Transplantation *64*, 1550–1558.

Dekel, B., Amariglio, N., Kaminski, N., Schwartz, A., Goshen, E., Arditti, F.D., Tsarfaty, I., Passwell, J.H., Reisner, Y., and Rechavi, G. (2002). Engraftment and differentiation of human metanephroi into functional mature nephrons after transplantation into mice is accompanied by a profile of gene expression similar to normal human kidney development. J. Am. Soc. Nephrol. *13*, 977–990.

Dekel, B., Burakova, T., Arditti, F.D., Reich-Zeliger, S., Milstein, O., Aviel-Ronen, S., Rechavi, G., Friedman, N., Kaminski, N., Passwell, J.H., et al. (2003). Human and porcine early kidney precursors as a new source for transplantation. Nat. Med. *9*, 53–60.

Dekel, B., Shezen, E., Even-Tov-Friedman, S., Katchman, H., Margalit, R., Nagler, A., and Reisner, Y. (2006a). Transplantation of human hematopoietic stem cells into ischemic and growing kidneys suggests a role in vasculogenesis but not tubulogenesis. Stem Cells *24*, 1185–1193.

Dekel, B., Metsuyanim, S., Schmidt-Ott, K.M., Fridman, E., Jacob-Hirsch, J., Simon, A., Pinthus, J., Mor, Y., Barasch, J., Amariglio, N., et al. (2006b). Multiple imprinted and stemness genes provide a link between normal and tumor progenitor cells of the developing human kidney. Cancer Res. *66*, 6040–6049.

Di Modugno, F., Iapicca, P., Boudreau, A., Mottolese, M., Terrenato, I., Perracchio, L., Carstens, R.P., Santoni, A., Bissell, M.J., and Nistico, P. (2012). Splicing program of human MENA produces a previously undescribed isoform associated with invasive, mesenchymal-like breast tumors. Proc. Natl. Acad. Sci. USA *109*, 19280–19285.

Duffield, J.S., Park, K.M., Hsiao, L.L., Kelley, V.R., Scadden, D.T., Ichimura, T., and Bonventre, J.V. (2005). Restoration of tubular epithelial cells during repair of the postischemic kidney occurs independently of bone marrow-derived stem cells. J. Clin. Invest. *115*, 1743–1755.

Dziedzic, K., Pleniceanu, O., and Dekel, B. (2014). Kidney stem cells in development, regeneration and cancer. Semin. Cell Dev. Biol. *36*, 57–65.

Harari-Steinberg, O., Pleniceanu, O., and Dekel, B. (2011). Selecting the optimal cell for kidney regeneration: fetal, adult or reprogrammed stem cells. Organogenesis *7*, 123–134.

Harari-Steinberg, O., Metsuyanim, S., Omer, D., Gnatek, Y., Gershon, R., Pri-Chen, S., Ozdemir, D.D., Lerenthal, Y., Noiman, T., Ben-Hur, H., et al. (2013). Identification of human nephron progenitors capable of generation of kidney structures and functional repair of chronic renal disease. EMBO Mol. Med. *5*, 1556–1568.

Harding, S.D., Armit, C., Armstrong, J., Brennan, J., Cheng, Y., Haggarty, B., Houghton, D., Lloyd-MacGilp, S., Pi, X., Roochun, Y., et al. (2011). The GUDMAP database—an online resource for genitourinary research. Development *138*, 2845–2853.

Kobayashi, A., Valerius, M.T., Mugford, J.W., Carroll, T.J., Self, M., Oliver, G., and McMahon, A.P. (2008). Six2 defines and regulates a multipotent self-renewing nephron progenitor population throughout mammalian kidney development. Cell Stem Cell *3*, 169–181.

Krause, D., and Cantley, L.G. (2005). Bone marrow plasticity revisited: protection or differentiation in the kidney tubule? J. Clin. Invest. *115*, 1705–1708.

Li, Z., Araoka, T., Wu, J., Liao, H.K., Li, M., Lazo, M., Zhou, B., Sui, Y., Wu, M.Z., Tamura, I., et al. (2016). 3D culture supports long-term expansion of mouse and human nephrogenic progenitors. Cell Stem Cell *19*, 516–529.

Little, M., Georgas, K., Pennisi, D., and Wilkinson, L. (2010). Kidney development: two tales of tubulogenesis. Curr. Top. Dev. Biol. *90*, 193–229.

Metsuyanim, S., Harari-Steinberg, O., Buzhor, E., Omer, D., Pode-Shakked, N., Ben-Hur, H., Halperin, R., Schneider, D., and Dekel, B. (2009). Expression of stem cell markers in the human fetal kidney. PLoS One *4*, e6709.

Morizane, R., Lam, A.Q., Freedman, B.S., Kishi, S., Valerius, M.T., and Bonventre, J.V. (2015). Nephron organoids derived from human pluripotent stem cells model kidney development and injury. Nat Biotechnol. *33* (11), 1193–1200.

O'Brien, L.L., Guo, Q., Lee, Y., Tran, T., Benazet, J.D., Whitney, P.H., Valouev, A., and McMahon, A.P. (2016). Differential regulation of mouse and human nephron progenitors by the Six family of transcriptional regulators. Development *143*, 595–608.

Pignatelli, J., Goswami, S., Jones, J.G., Rohan, T.E., Pieri, E., Chen, X., Adler, E., Cox, D., Maleki, S., Bresnick, A., et al. (2014). Invasive breast carcinoma cells from patients exhibit MenaINV- and macrophage-dependent transendothelial migration. Sci. Signal. *7*, ra112.

Pleniceanu, O., Harari-Steinberg, O., and Dekel, B. (2010). Concise review: kidney stem/progenitor cells: differentiate, sort out, or reprogram? Stem Cells *28*, 1649–1660.

Pode-Shakked, N., Metsuyanim, S., Rom-Gross, E., Mor, Y., Fridman, E., Goldstein, I., Amariglio, N., Rechavi, G., Keshet, G., and Dekel, B. (2009). Developmental tumourigenesis: NCAM as a putative marker for the malignant renal stem/progenitor cell population. J. Cell. Mol. Med. *13*, 1792–1808.

Pode-Shakked, N., Shukrun, R., Mark-Danieli, M., Tsvetkov, P., Bahar, S., Pri-Chen, S., Goldstein, R.S., Rom-Gross, E., Mor, Y., Fridman, E., et al. (2013). The isolation and characterization of renal cancer initiating cells from human Wilms' tumour xenografts unveils new therapeutic targets. EMBO Mol. Med. *5*, 18–37.

Pode-Shakked, N., Pleniceanu, O., Gershon, R., Shukrun, R., Kanter, I., Bucris, E., Pode-Shakked, B., Tam, G., Tam, H., Caspi, R., et al. (2016). Dissecting stages of human kidney development and tumorigenesis with surface markers affords simple prospective purification of nephron stem cells. Sci. Rep. *6*, 23562.

Rinkevich, Y., Montoro, D.T., Contreras-Trujillo, H., Harari-Steinberg, O., Newman, A.M., Tsai, J.M., Lim, X., Van-Amerongen, R.,

Bowman, A., Januszyk, M., et al. (2014). In vivo clonal analysis reveals lineage-restricted progenitor characteristics in mammalian kidney development, maintenance, and regeneration. Cell Rep. *7*, 1270–1283.

Shapiro, I.M., Cheng, A.W., Flytzanis, N.C., Balsamo, M., Condeelis, J.S., Oktay, M.H., Burge, C.B., and Gertler, F.B. (2011). An EMT-driven alternative splicing program occurs in human breast cancer and modulates cellular phenotype. PLoS Genet. *7*, e1002218.

Shukrun, R., Pode-Shakked, N., Pleniceanu, O., Omer, D., Vax, E., Peer, E., Pri-Chen, S., Jacob, J., Hu, Q., Harari-Steinberg, O., et al. (2014). Wilms' tumor blastemal stem cells dedifferentiate to propagate the tumor bulk. Stem Cell Reports *3*, 24–33.

Takasato, M., Er, P.X., Chiu, H.S., Maier, B., Baillie, G.J., Ferguson, C., Parton, R.G., Wolvetang, E.J., Roost, M.S., Chuva de Sousa Lopes, S.M., and Little, M.H. (2015). Kidney organoids from human iPS cells contain multiple lineages and model human nephrogenesis. Nature *526*, 564–568.

Tanigawa, S., Taguchi, A., Sharma, N., Perantoni, A.O., and Nishinakamura, R. (2016). Selective In Vitro Propagation of Nephron Progenitors Derived from Embryos and Pluripotent Stem Cells. Cell Rep. *15* (4), 801–813.

Trivedi, H. (2010). Cost implications of caring for chronic kidney disease: are interventions cost-effective? Adv. Chronic Kidney Dis. *17*, 265–270.

Vivante, A., Mark-Danieli, M., Davidovits, M., Harari-Steinberg, O., Omer, D., Gnatek, Y., Cleper, R., Landau, D., Kovalski, Y., Weissman, I., et al. (2013). Renal hypodysplasia associates with a WNT4 variant that causes aberrant canonical WNT signaling. J. Am. Soc. Nephrol. *24*, 550–558.

Stem Cell Reports, Volume *9*

Supplemental Information

Evidence of In Vitro Preservation of Human Nephrogenesis at the Single-Cell Level

Naomi Pode-Shakked, Rotem Gershon, Gal Tam, Dorit Omer, Yehudit Gnatek, Itamar Kanter, Sarit Oriel, Guy Katz, Orit Harari-Steinberg, Tomer Kalisky, and Benjamin Dekel

EVIDENCE OF IN VITRO PRESERVATION OF HUMAN NEPHROGENESIS AT THE SINGLE CELL LEVEL

Pode-Shakked Naomi[1,2,3,7,8], Gershon Rotem[1,2,7,8], Tam Gal[4], Omer Dorit[1,2,] , Gnatek Yehudit[1,2], Kanter Itamar[4], Oriel Sarit[4], Katz Guy[1,2,3,5,7], Harari-Steinberg Orit[1,2], Kalisky Tomer[4,9], Dekel Benjamin[1,2,6,7, 9#]

[1]Pediatric Stem Cell Research Institute, Edmond and Lily Safra Children's Hospital, Sheba Medical Center, Tel-Hashomer, Israel

[2]Sheba Centers for Regenerative Medicine and Cancer Research, Sheba Medical Center, Tel-Hashomer, Israel

[3]The Dr. Pinchas Borenstein, Talpiot Medical Leadership Program, Sheba Medical Center, Tel-Hashomer, Israel

[4]Faculty of Engineering and Bar-Ilan Institute of Nanotechnology and Advanced Materials (BINA), Bar-Ilan University, Ramat Gan, Israel.

[5]The Joseph Buchman Gynecology and Maternity Center,Sheba Medical Center, Tel-Hashomer, Israel

[6]Division of Pediatric Nephrology, Edmond and Lily Safra Children's Hospital,

Sheba Medical Center, Tel-Hashomer, Israel

[7]Sackler Faculty of Medicine, Tel-AvivUniversity, Tel-Aviv, Israel

[8]The first two authors share first co-authorship

[9]The last two authors share senior co-authorship

Supplemental Information table of contents:

SUPPLEMENTAL FIGURES AND TABLES 3

FIGURE S1| SIX2 AND EPCAM EXPRESSION IN HFK CELLS GROWN IN MNPEM REVEALS CHARACTERISTIC "NICHES" 3

FIGURE S2| FACS ANALYSIS OF HFK ACCORDING TO NCAM, CD133, AND EPCAM EXPRESSION LEVELS SHOWS THREE CELL POPULATIONS AT DIFFERENT STAGES OF DIFFERENTIATION WITHIN THE NCAM+CD133- FRACTION 4

FIGURE S3| IDENTIFICATION OF THE SECOND (NON CAP) MESENCHYMAL CELL POPULATION. SINGLE CELL QPCR GENE EXPRESSION ANALYSIS OF HFK CELLS GROWN IN SFM AND SORTED ACCORDING TO NCAM AND CD133 SHOWS A PREDOMINANTLY EPITHELIAL PHENOTYPE..5

FIGURE S4| NCAM+CD133-EPCAM- CELLS GROWN IN MNPEM PRESERVE SIX2 EXPRESSION AND CAN DIFFERENTIATE TO PROXIMAL AND DISTAL TUBULAR EPITHELIA. FOXD1 IS DOWNREGULATED IN HFK CELLS GROWN IN MNPEM. 7

TABLE S1| 48 TAQMAN GENE EXPRESSION ASSAYS USED FOR "BULK" MICROFLUIDIC MULTIPLEXED QPCR......8

TABLE S2| AVERAGE PERCENTAGES (N=3) OF NEPHROGENIC RENAL MET COMPARTMENTS ACCORDING TO NCAM, CD133, AND EPCAM IN FRESH HFK AND HFK GROWN IN MNPEM, SCM AND SFM. 10

TABLE S3| 48 TAQMAN GENE EXPRESSION ASSAYS USED FOR SINGLE CELL MICROFLUIDIC MULTIPLEXED QPCR 11

SUPPLEMENTAL EXPERIMENTAL PROCEDURES 13

IF STAINING OF CELLS 13

IN VIVO WT XENOGRAFT FORMATION...13

SINGLE CELL GENE EXPRESSION ANALYSIS 13

STATISTICAL ANALYSIS...14

REFERENCES.. 15

Supplemental figures and tables

Figure S1| SIX2 and EpCAM expression in hFK cells grown in mNPEM reveals characteristic "niches"

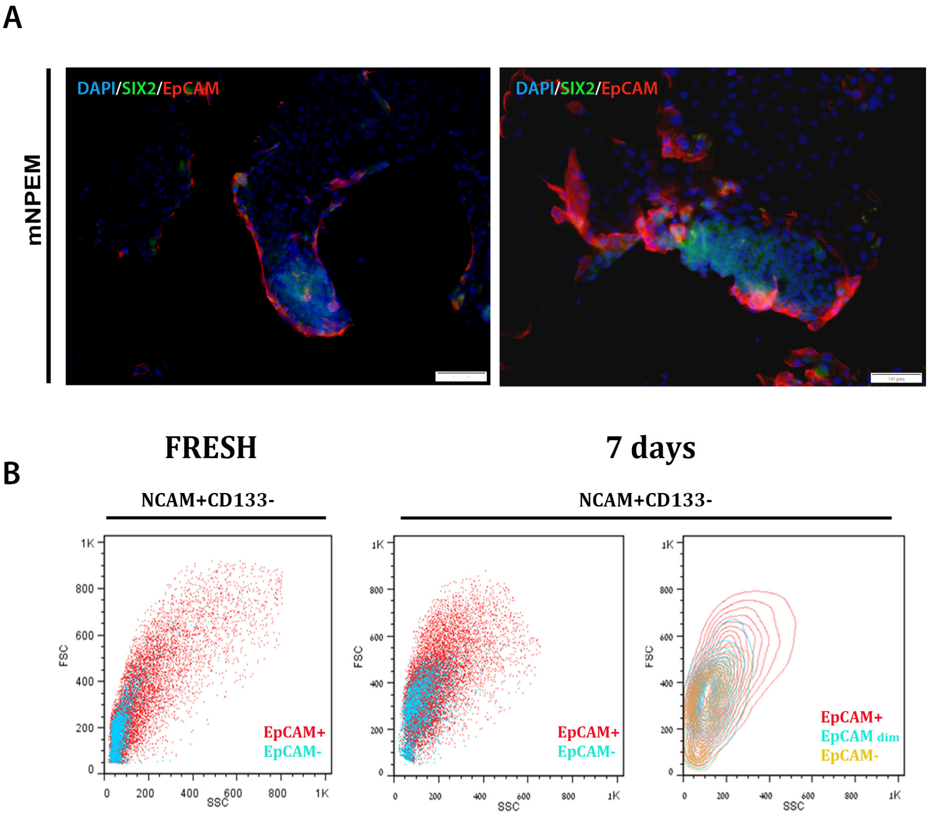

(A) Double labeling of hFK cells grown in mNPEM for SIX2 and EpCAM reveals a unique "niche" microstructure characterized by small inner cells that positively stain for SIX2 and peripheral larger epithelial cells that positively stain for EpCAM. Images were obtained using Olympus DP72 camera attached to Olympus BX51 fluorescence microscope and processed via cell Sens standard software. Bar represents 100µm in left panel and 50µm in right panel; (B) Representative FACS analysis of Fresh hFK and hFK cultured in mNPEM for 7 days based on forward and side scatter (FSC/SSC) plot. Close examination of the different cell subpopulations according to EpCAM distribution within the NCAM1+CD133- cell fraction shows EpCAM- (as well as EpCAM-/dim) cells in both Fresh hFK and mNPEM cultured hFK to possess low FSC and SSC parameters relative to EpCAM+ (as well as EpCAMbright), in accordance with their small size. These findings demonstrate that similar cellular diversity is found in both mNPEM-grown hFK cells and in fresh uncultured hFK cells. (Related to Figure 1)

Figure S2| FACS analysis of hFK according to NCAM, CD133, and EpCAM expression levels shows three cell populations at different stages of differentiation within the NCAM+CD133- fraction

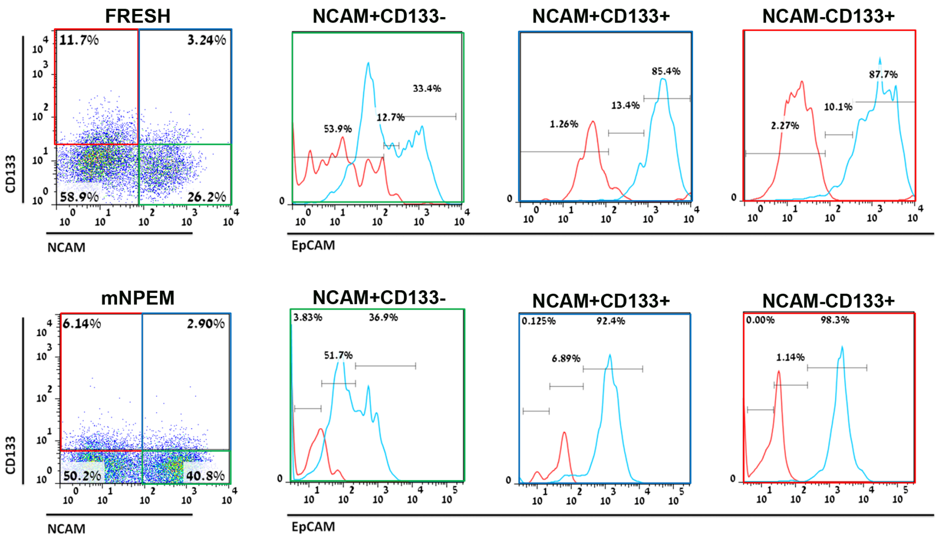

Representative FACS analysis of Fresh hFK and hFK cultured in mNPEM for 7 days (3rd hFK replicate). Cells grown in mNPEM preserves the NCAM1$^+$CD133$^-$ cell fraction in a way that is comparable to fresh cells (~26% and 40% respectively). Within the NCAM1$^+$CD133$^-$ cell fraction most cells are EpCAM^{dim} or EpCAM$^-$ in both Fresh hFK and hFK cultured in mNPEM (53.9+12.7=66.6% and 3.83+51.7=55.53% respectively). Moreover, the NCAM1$^+$CD133$^+$ and NCAM$^-$CD133$^+$ subpopulations show gradual increase in EpCAM^{bright} cells (92% and 98% respectively) in mNPEM grown cells. This is consistent with earlier findings and provides further support to our hypothesis that all hFK renal MET epithelial lineages are preserved in mNPEM (see also Figure 2C). (Related to Figure 2)

Figure S3| Identification of the second (non Cap) mesenchymal cell population. Single cell qPCR gene expression analysis of hFK cells grown in SFM and sorted according to NCAM and CD133 shows a predominantly epithelial phenotype

A

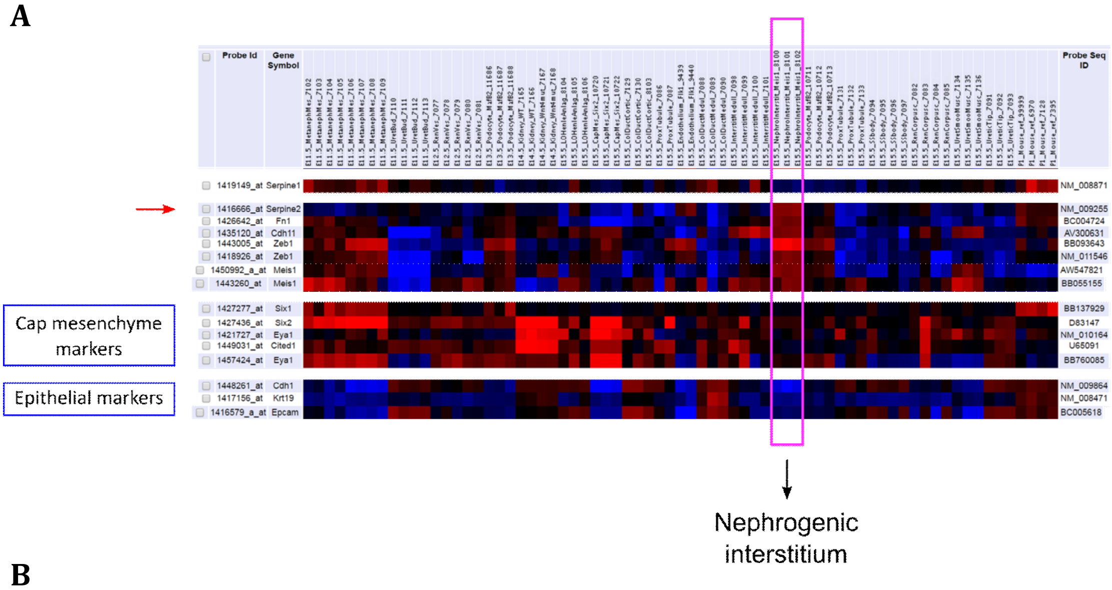

B

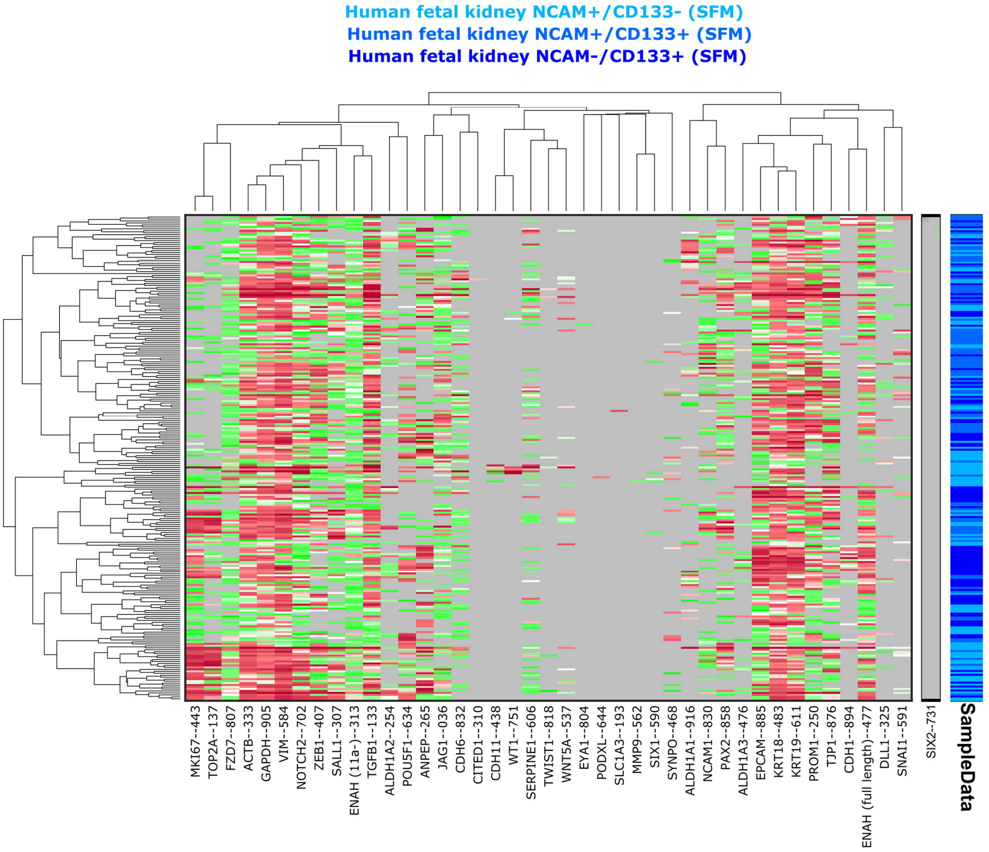

(A) Identification of the second (non-Cap) human mesenchymal cell population in hFK cells grown in mNPEM. Shown are microarray expression measurements queried from the GUDMAP database (http://www.gudmap.org). It can be seen that the nephrogenic interstitial (Meis1+) cell population in E15.5 mouse embryos [1] behaves similar to the second human non-Cap mesenchymal cell population, with SERPINE2 – a paralog of SERPINE1 – being over-expressed along with CDH11 and ZEB1. The Cap-mesenchyme markers (SIX1, SIX2, EYA1, and CITED1) and epithelial markers (CDH1, KRT19, and EpCAM) are relatively under-expressed in this population. We therefore hypothesize that the second non-Cap mesenchymal cell subpopulation (in hFK cells grown in mNPEM) represents the nephrogenic interstitium, and that SERPINE2 in mice interchanges with its paralog SERPINE1 in humans as a marker for the nephrogenic interstitial cells. (B) A gene expression heat map of ~240 single cells (rows) and 42 genes (columns) measured simultaneously from each cell. Gene expression levels (in terms of threshold cycles, Ct) were standardized and clustered such that phenotypically similar cells are grouped next to each other (Red – high expression, Green – low expression, Gray – no expression). Cells were isolated from human fetal kidney, cultured in SFM for 7 days, and sorted by FACS into 3 fractions: NCAM+CD133-, NCAM+CD133+, and NCAM-CD133+. The colors in the column on the right of the heatmap represent the FACS-sorted fraction-of-origin of each individual cell. It can be seen that all fractions have a predominantly epithelial phenotype (EPCAM+KRT18+KRT19+SIX2-). Although in the NCAM+CD133- fraction we did find a small number of more early mesenchymal cells (CDH11+), no measurable SIX2+ cells within the ~240 cells that we measured. Genes that had zero expression in all cells were excluded from the heatmap (apart from SIX2) (Related to Figure 3).

Figure S4| NCAM+CD133-EpCAM- cells grown in mNPEM preserve SIX2 expression and can differentiate to proximal and distal tubular epithelia. FOXD1 is downregulated in hFK cells grown in mNPEM.

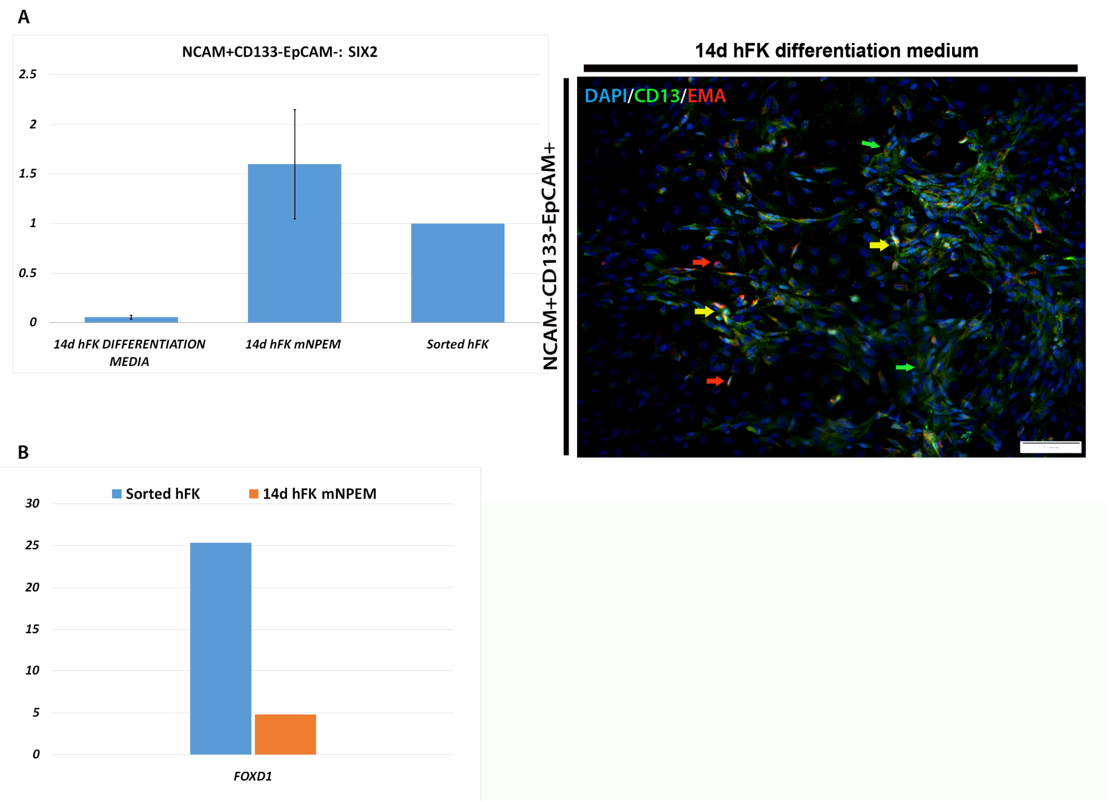

(A) Left panel: qRT-PCR analysis of SIX2 expression in hFK cells grown in mNPEM. NCAM1$^+$CD133$^-$EpCAM$^-$ cells cultured in mNPEM showed preservation of SIX2 expression after 14 days in culture that is comparable to freshly sorted hFK cells. In contrast, cells grown in a differentiation media show drastic downregulation of SIX2 expression. Note that the values for NCAM1$^+$CD133$^-$EpCAM$^-$ freshly sorted hFK cells were used as reference for normalization (therefore=1) and all other values were calculated with respect to them. Experiments were performed on hFK from 2 sources (n=2). Results are presented as the mean±S.E.M of three separate experiments; *$p<0.05$; Right panel: Immunofluorescence staining of NCAM1$^+$CD133$^-$EpCAM$^-$ cells grown in a differentiation media for 14 days for CD13 (a proximal tubule marker, green) and EMA (a distal tubule marker, red) demonstrating several stages of renal MET of human nephrogenesis by cells expressing either CD13 (green arrow) or EMA (red arrow). Double labeling for both CD13 and EMA for a small number of cells suggests an earlier intermediate state of epithelial differentiation (yellow arrow). (B) qRT-PCR analysis of FOXD1 expression in sorted hFK cells grown in mNPEM reveals dramatic downregulation in FOXD1 expression levels at day 14 suggesting the reduction of stromal elements (FOXD1$^+$) compared to T0. (Related to Figure 1).

Table S1| 48 Taqman gene expression assays used for "bulk" microfluidic multiplexed qPCR

	Assay ID	Gene Symbol
1	Hs00357333_g1	ACTB
2	Hs00946916_m1	ALDH1A1
3	Hs00174265_m1	ANPEP
4	Hs00166067_m1	AQP1
5	Hs00185020_m1	AQP3
6	Hs00984230_m1	B2M
7	Hs01023894_m1	CDH1
8	Hs00191832_m1	CDH6
9	Hs00366310_m1	CITED1
10	Hs00153607_m1	CUBN
11	Hs01011325_g1	DLL1
12	Hs00901885_m1	EPCAM
13	Hs00559473_s1	FOXC1
14	Hs00270117_s1	FOXD1
15	Hs99999905_m1	GAPDH
16	Hs00735523_m1	IRX3
17	Hs01070036_m1	JAG1
18	Hs00969422_m1	LGR5
19	Hs00189742_m1	LRP2
20	Hs01032443_m1	MKI67
21	Hs00410317_m1	MUC1
22	Hs00941830_m1	NCAM1
23	Hs01050702_m1	NOTCH2

24	Hs00190446_m1	NPHS1
25	Hs00377071_m1	OSR1
26	Hs00240858_m1	PAX2
27	Hs00248563_m1	PLCB1
28	Hs01574644_m1	PODXL
29	Hs01009250_m1	CD133
30	Hs00230907_m1	RARA
31	Hs00231307_m1	SALL1
32	Hs01548617_m1	SCNN1B
33	Hs00232731_m1	SIX2
34	Hs00165731_m1	SLC12A1
35	Hs01027568_m1	SLC12A3
36	Hs01573790_m1	SLC5A1
37	Hs00195591_m1	SNAI1
38	Hs00950344_m1	SNAI2
39	Hs00998133_m1	TGFB1
40	Hs01032137_m1	TOP2A
41	Hs01675818_s1	TWIST1
42	Hs00358451_m1	UMOD
43	Hs00185584_m1	VIM
44	Hs00260769_m1	WNK4
45	Hs01055707_m1	WNT3A
46	Hs00229142_m1	WNT4
47	Hs00998537_m1	WNT5A
48	Hs01103751_m1	WT1

(Related to Figure 1)

Table S2| Average percentages (n=3) of nephrogenic renal MET compartments according to NCAM, CD133, and EpCAM in fresh hFK and hFK grown in mNPEM, SCM and SFM.

	FRESH	mNPEM	SCM	SFM
NCAM+CD133-	20.05	20.33	4.00	3.02
NCAM+CD133-EPCAM-	5.51	7.42	0.14	0.13
NCAM+CD133-EPCAMdim	6.82	5.74	0.98	0.42
NCAM+CD133-EPCAMbright	6.92	7.52	2.88	2.45

(Related to Figure 2)

Table S3| 48 Taqman gene expression assays used for single cell microfluidic multiplexed qPCR

	Assay ID	Gene symbol
1	Hs00357333_g1	ACTB
2	Hs00946916_m1	ALDH1A1
3	Hs00180254_m1	ALDH1A2
4	Hs00167476_m1	ALDH1A3
5	Hs00174265_m1	ANPEP
6	Hs01023894_m1	CDH1
7	Hs00156438_m1	CDH11
8	Hs00191832_m1	CDH6
9	Hs00366310_m1	CITED1
10	Hs01011325_g1	DLL1
11	Hs04260477_m1	ENAH (full length)
12	Hs00983313_g1	ENAH (11a-)
13	Hs00901885_m1	EPCAM
14	Hs00166804_m1	EYA1
15	Hs00942807_s1	FZD7
16	Hs99999905_m1	GAPDH
17	Hs01070036_m1	JAG1
18	Hs02827483_g1	KRT18
19	Hs01051611_gH	KRT19
20	Hs00412974_m1	MFAP4
21	Hs01032443_m1	MKI67
22	Hs00957562_m1	MMP9
23	Hs00410317_m1	MUC1

24	Hs00941830_m1	NCAM1
25	Hs01050702_m1	NOTCH2
26	Hs00377071_m1	OSR1
27	Hs00240858_m1	PAX2
28	Hs01574644_m1	PODXL
29	Hs00999634_gH	POU5F1
30	Hs01009250_m1	CD133
31	Hs00231307_m1	SALL1
32	Hs00413788_m1	SALL2
33	Hs01126606_m1	SERPINE1
34	Hs00195590_m1	SIX1
35	Hs00232731_m1	SIX2
36	Hs00188193_m1	SLC1A3
37	Hs00195591_m1	SNAI1
38	Hs00950344_m1	SNAI2
39	Hs00702468_s1	SYNPO
40	Hs00998133_m1	TGFB1
41	Hs01551876_m1	TJP1
42	Hs01032137_m1	TOP2A
43	Hs01675818_s1	TWIST1
44	Hs00185584_m1	VIM
45	Hs00229142_m1	WNT4
46	Hs00998537_m1	WNT5A
47	Hs01103751_m1	WT1
48	Hs01566407_m1	ZEB1

(Related to Figure 3)

Supplemental experimental procedures

IF staining of cells

Cells were fixed with 4% PFA in PBS for 10 min, and washed with PBS–Tween (0.05%). Then, the cells were blocked with Cas-Block solution for 1 h at RT followed by incubation with primary antibodies for SIX2 (11562-1-AP, Proteintech), EpCAM (CBL251,Millipore), CD13 (Sigma), and EMA (Cell Marque) for 1h in room temperature. Cells were then washed and then incubated with a secondary antibody for 1h in room temperature. Following PBS-Tween washes, mounting containing DAPI (DapiFluoromount-G; SouthernBiotech, 0100-20) was applied. Images were obtained by Olympus BX51TF fluorescence microscope using Olympus DP72 camera and cellSens standard software. For comparative analysis between the fluorescence signal of SIX2 cells, images were analyzed using ImageJ software. Signal quantification was calculated for each nucleus by determining its area and quantification of the signal in that area. The corrected total cell fluorescence (CTCF) was calculated as: integrated density − (area of selected cell × mean fluorescence of background reading).

In vivo WT xenograft formation

All animal experiments were conducted in accordance with the National Institutes of Health guidelines for the care and use of animals and with an approved animal protocol from the Sheba medical center Animal Care and Use Committee. Initial WT xenografting to 5-8 weeks old, female, nonobese diabetic immune-deficient mice was performed as previously described [2]. Briefly, primary WT tissue was cut into 2-5mm pieces and implanted subcutaneously in the back of the mouse. Tumors were harvested approximately 3-6 month post implantation or when they reached a size of 1.5cm diameter. Single cells suspensions were obtained by mincing the samples in Iscove's modification of Dulbecco's medium (IMDM) containing antibiotics (penicillin and streptomycin), followed by treatment with collagenase IV for 2h at 37◦C. After addition of IMDM at twice the volume of the collagenase solution, the enzymatically treated tissue was triturated and the suspension was filtered using a 100µm cell strainer and washed twice with IMDM containing antibiotics. Erythrocytes were removed by ACK RBS lysis buffer.

Single cell gene expression analysis

We combined single cell expression data from the following biological samples:

1. Human fetal kidney no. 104 (hFK104): Cells isolated from a human fetal kidney, cultured for 7 days in NPEM for a single passage, and sorted by FACS into 3 fractions: $NCAM^{+}CD133^{-}EpCAM^{-}$, $NCAM^{+}CD133^{-}EpCAM^{dim}$, and $NCAM^{+}CD133^{+}$.

2. Human fetal kidney no. 103 (hFK103): Cells isolated from a human fetal kidney, cultured for 7 days in SFM for a single passage, and sorted by FACS into 3 fractions: $NCAM^+CD133^-$, $NCAM^+CD133^+$, $NCAM^-CD133^+$. Additionally, a 4th fraction of non-gated cells (ignoring markers) was sorted.

3. Wilms' tumor no. 11 – a patient-derived xenograft (W011 PDX): A late passage blastemal Wilms' tumor patient-derived xenograft. After 10 passages in NOD-SCID mice, cells were cultured for 4 days in SCM, and sorted into 2 fractions: $NCAM^+ALDH1^+$ (the tumorigenic fraction) and $NCAM^+ALDH1^-$ (the non-tumorigenic fraction).

Statistical analysis

Results are expressed as the mean ± S.E.M, unless otherwise indicated. Statistical differences in gene expression between hFK cell populations were evaluated using the non-parametric, one sided Sign test. Statistical differences between additional data groups were determined with Student's t test. For all statistical analysis, the level of significance was set as $p<0.05$ unless otherwise indicated. All experiments were performed in duplicates and on at least 3 tissue sources (n=3).

References

1. Brunskill EW, A.B., Georgas K, Rumballe B, Valerius MT, Aronow J, et al. , *Atlas of Gene Expression in the Developing Kidney at Microanatomic Resolution. .* Dev Cell., 2008. **15**: p. 781–791.

2. Dekel, B., et al., Multiple imprinted and stemness genes provide a link between normal and tumor progenitor cells of the developing human kidney. Cancer Res, 2006. **66**(12): p. 6040-9.
